# Supplementary material for: Neural relational inference to learn long-range allosteric interactions in proteins from molecular dynamics simulations
Source: Nat Commun. 2022 Mar 29;13:1661. doi: 10.1038/s41467-022-29331-3 (PMC8964751; doi:10.1038/s41467-022-29331-3)
Supplement: Supplementary file 1 — Supplementary information [file 41467_2022_29331_MOESM1_ESM.pdf]

Supplementary Information of
Neural relational inference to learn long-range allosteric interactions in proteins from molecular dynamics simulations

Jingxuan Zhu[1, 2, #], Juexin Wang[2, #], Weiwei Han[1, *], Dong Xu[2, *]
[1] Key Laboratory for Molecular Enzymology and Engineering of Ministry of Education, School of Life Science, Jilin University, Changchun, China
[2] Department of Electrical Engineering and Computer Science, Bond Life Sciences Center, University of Missouri, Columbia, Missouri, United States
# These authors contributed equally to the paper as first authors
* To whom correspondence should be addressed
Dr. Weiwei Han. Email: weiweihan@jlu.edu.cn;
Dr. Dong Xu. Email: xudong@missouri.edu

Supplementary Figures

Supplementary Figure 1: Stability profile of Pin1 system (PDB ID: 3TDB).
Supplementary Figure 2: Comparison of RMSF values between truth and reconstruction of trajectories for Pin1 system.
Supplementary Figure 3: Frequency of each residue on its shortest pathways for two forms of FFpSPR-bound Pin1.
Supplementary Figure 4: The average structures of FFpSPR- and pCdc25C-bound Pin1 represent the top 6 most populated clusters obtained from MD trajectories.
Supplementary Figure 5: RMSF plot of SOD1 system.
Supplementary Figure 6: Relevant distance distributions between atoms that form hydrogen bond interactions in the trajectories of SOD1 system.
Supplementary Figure 7: SOD1 structure containing residues that form hydrogen bond interactions.
Supplementary Figure 8: Rg plot of SOD1 system.
Supplementary Figure 9: Comparison of RMSF values between truth and reconstruction of trajectories for SOD1 system.
Supplementary Figure 10: Secondary structure analysis for MEK1 system.
Supplementary Figure 11: Principal component analysis (PCA) for MEK1 system.
Supplementary Figure 12: Comparison of RMSF values between truth and reconstruction of trajectories for MEK1 system.
Supplementary Figure 13: Frequency of each residue on the shortest pathways for S218Sp/S222Sp and E203K of MEK1.
Supplementary Figure 14: Mean squared error (MSE) and value square deviation (VSD) for Pin1, SOD1, and MEK1 systems.
Supplementary Figure 15: Clustering results of Pin1, SOD1, and MEK1 systems.
Supplementary Figure 16: The distribution of learned edges for Pin1 system (PDB ID: 3TDB).
Supplementary Figure 17: The distribution of learned edges for Pin1 system (PDB ID: 1NMV).
Supplementary Figure 18: The distribution of learned edges for SOD1 system.
Supplementary Figure 19: The distribution of learned edges for MEK1 system.

Supplementary Figure 20: Comparison of RMSF values between the simulation and the reconstruction of trajectories for SOD1 system.
Supplementary Figure 21: The distribution of learned edges for SOD1 system.
Supplementary Figure 22: Comparison of RMSF values between the simulation and the reconstruction of trajectories for the Apo-Pin1, FFpSPR-bound Pin1, and FFpSPR-bound Pin1 (I28A). The total simulation time is 500 ns.
Supplementary Figure 23: The distribution of learned edges for the Apo-Pin1, FFpSPR-bound Pin1, and FFpSPR-bound Pin1 (I28A). The total simulation time is 500 ns.
Supplementary Figure 24: Comparison of RMSF values between the simulation and the reconstruction of trajectories for FFpSPR-bound Pin1 with two domains-separated, and pCdc25C-bound Pin1 with two domains-separated.
Supplementary Figure 25: The distribution of learned edges for FFpSPR-bound Pin1 with two domains-separated, and pCdc25C-bound Pin1 with two domains-separated.
Supplementary Figure 26: Comparison of RMSF values between the simulation and the reconstruction of trajectories for the Apo-Pin1, FFpSPR-bound Pin1, and FFpSPR-bound Pin1 (I28A). The total simulation time is 200 ns.
Supplementary Figure 27: The distribution of learned edges for the Apo-Pin1, FFpSPR-bound Pin1, and FFpSPR-bound Pin1 (I28A). The total simulation time is 200 ns.
Supplementary Figure 28: The distribution of learned edges for three repeated trajectories of the Pin1, SOD1, and MEK1 systems.
Supplementary Figure 29: The node centralities in the allosteric pathways between the WW domain and the catalytic loop for three repeated trajectories of FFpSPR-Pin1 complexes.
Supplementary Figure 30: The node centralities in the allosteric pathways between residue G93/A93 and the electrostatic loop for three repeated trajectories of WT- and G93A-SOD1 complexes.
Supplementary Figure 31: The node centralities in the allosteric pathways between the activation segment and the αC-helix/proline-rich loop for three repeated trajectories of S218Sp/S222Sp- and E203K-MEK1 complexes.
Supplementary Figure 32: Comparison of mean squared error (MSE) values between the proposed model and the model without latent variables on edges.
Supplementary Figure 33: The node centralities in the allosteric pathways between the WW domain and the catalytic loop for the WT and 23 Ala-mutants of unbound Pin1.
Supplementary Figure 34: Residue-residue interaction maps obtained by the NRI model for the WT and 23 Ala-mutants of unbound Pin1.
Supplementary Figure 35: Comparison of residue-residue interaction maps obtained by constraint network analysis (CNA) and the GNN-based NRI model.
Supplementary Figure 36: The distribution of learned edges and the correlation analysis for Pin1 system (PDB ID: 1NMV). The edges are learned from frames 1-1000, 1000-2000, …, 4000-5000 of trajectories.
Supplementary Figure 37: The distribution of learned edges between residues for Pin1 system (PDB ID: 1NMV). The edges are learned from frames 1-500, 1-1000, …, 1-5000 of trajectories.
Supplementary Figure 38: Correlation analysis for Pin1 system (PDB ID: 1NMV). The edges are learned from frames 1-500, 1-1000, …, 1-5000 of trajectories.

Supplementary Figure 39: Comparison of dynamics coupling index (DCI) analysis and the NRI-based approach.
Supplementary Figure 40: The node centralities in the allosteric pathways between the WW domain and the catalytic loop for Pin1 system.
Supplementary Figure 41: The allosteric pathways mapped on the Pin1 structures for Pin1 system.
Supplementary Figure 42: The node centralities in the allosteric pathways between the G93/A93 and the electrostatic loop for SOD1 system.
Supplementary Figure 43: The allosteric pathways mapped on the SOD1 structures for the SOD1 system.
Supplementary Figure 44: The node centralities in the allosteric pathways between the activation segment and the αC-helix/proline-rich loop for the MEK1 system.
Supplementary Figure 45: The allosteric pathways mapped on the MEK1 structures for MEK1 system.

Supplementary Tables

Supplementary Table 1: Shortest pathways for Pin1 system
Supplementary Table 2: Probability of hydrogen-bond formation in the trajectories of WT and G93A of SOD1
Supplementary Table 3: Shortest pathways for SOD1 system
Supplementary Table 4: Shortest pathways for MEK1 system
Supplementary Table 5: Mean squared error (MSE) values for the proposed model and the model without latent variables on edges.

Supplementary Notes

Supplementary Note 1: Simulation data
Supplementary Note 2: NRI model construction details
Supplementary Note 3: Free energy quantity derived from mechanical stability (Constraint network analysis)
Supplementary Note 4: Derivative centrality metric of the Hessian
Supplementary Note 5: Dynamic coupling index (DCI)
Supplementary Note 6: Free energy score derived from the NRI modeling

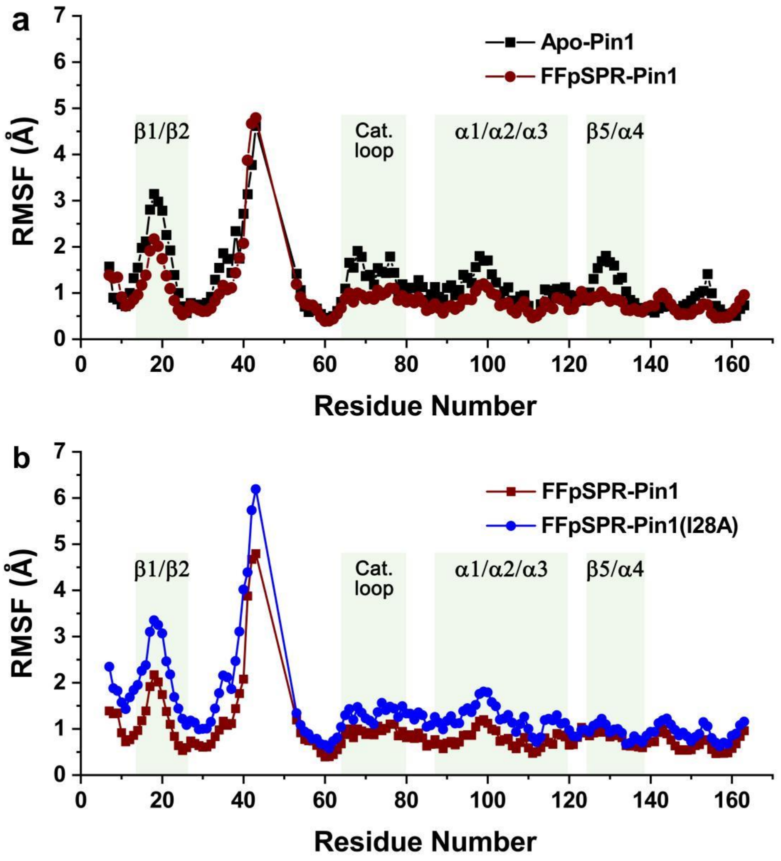

Supplementary Figure 1: Stability profile of Pin1 system (PDB ID: 3TDB). Comparison of RMSF values between apo Pin1 and FFpSPR-bound Pin1 (**a**). Comparison of RMSF values between FFpSPR-bound Pin1 and FFpSPR-bound Pin1 (I28A) (**b**). The domains presented here are β1-2 sheets in the WW domain (β1/2), catalytic loop (Cata. loop), α1-3 helices (α1/α2/α3), and β5-sheet/α4-helix in the PPIase core (β5/α4).

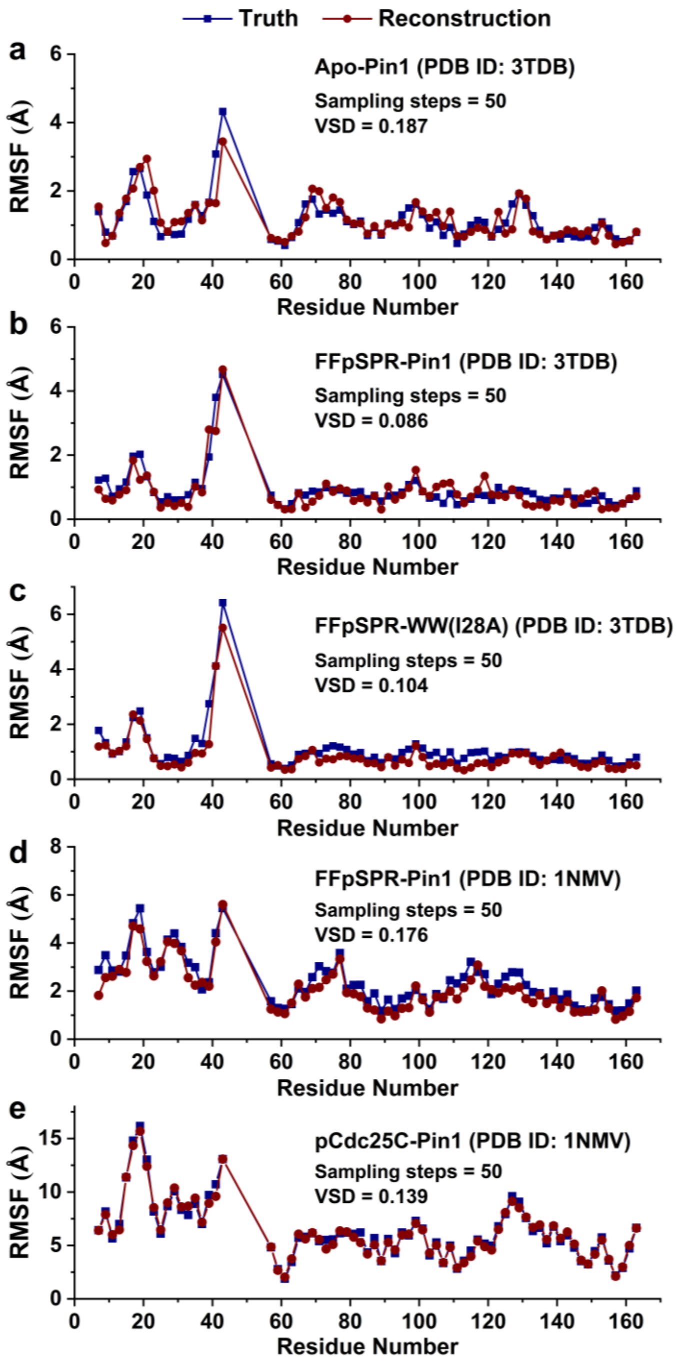

Supplementary Figure 2: Comparison of RMSF values between truth and reconstruction of trajectories for apo Pin1 (**a**), FFpSPR-bound Pin1 (**b**), FFpSPR-bound Pin1 (I28A) (**c**), FFpSPR-bound Pin1 with two domains-separated (**d**), and pCdc25C-bound Pin1 with two domains-separated (**e**). The reconstructed trajectories were obtained based on 50 sampling steps. The value square deviation (VSD), on average, 0.086~0.187, demonstrates that the model is able to correctly reconstruct the trajectories of Pin1 system using 50 sampling steps.

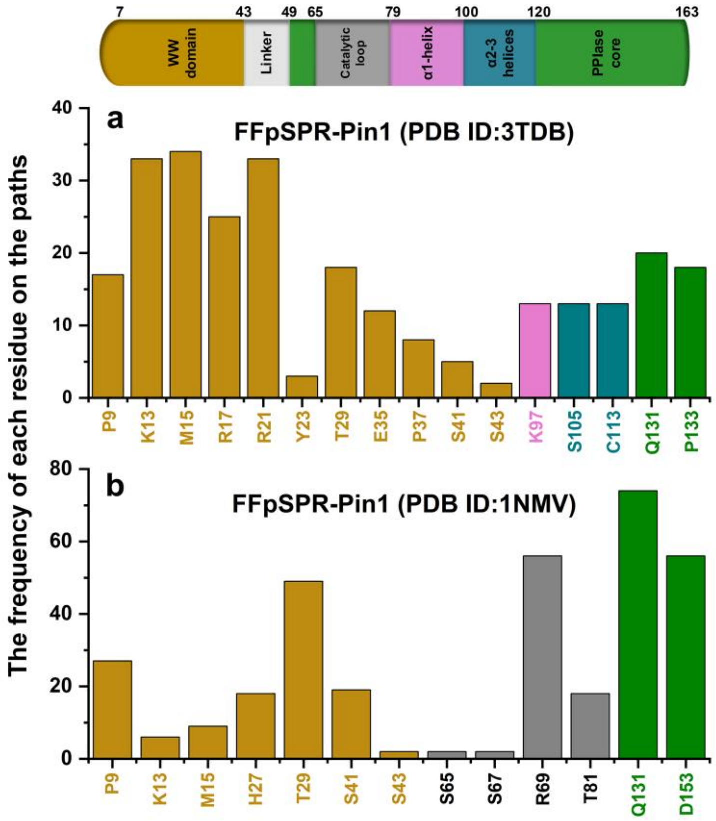

Supplementary Figure 3: Frequency of each residue on its shortest pathways for FFpSPR-bound Pin1 (PDB ID: 3TDB) (**a**) and FFpSPR-bound Pin1 with the WW domain and the PPIase core separated (PDB ID: 1NMV) (**b**). The bar colors indicate protein domains. Other residues not shown have no or few occurrences on the shortest pathways.

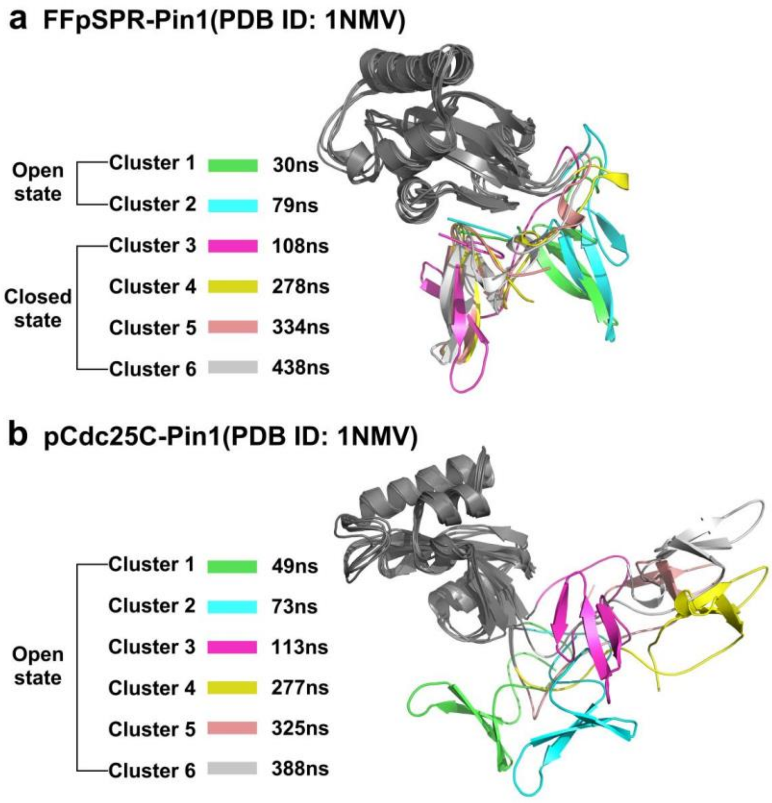

Supplementary Figure 4: The average structures of FFpSPR-bound Pin1 (**a**) and pCdc25C-bound Pin1(**b**) represent the top 6 most populated clusters obtained from MD trajectories.

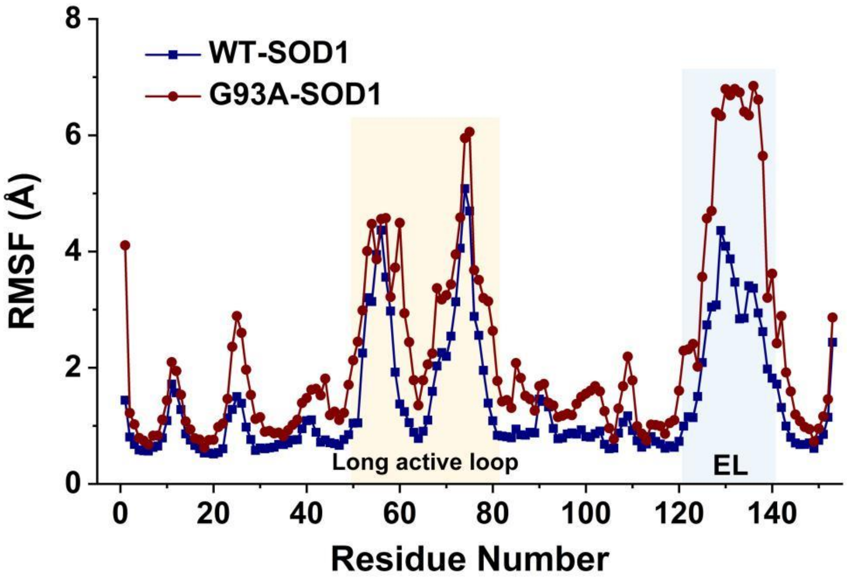

Supplementary Figure 5: RMSF plot of WT SOD1 and G93A SOD1 systems. The domain presented here is Electrostatic loop (EL).

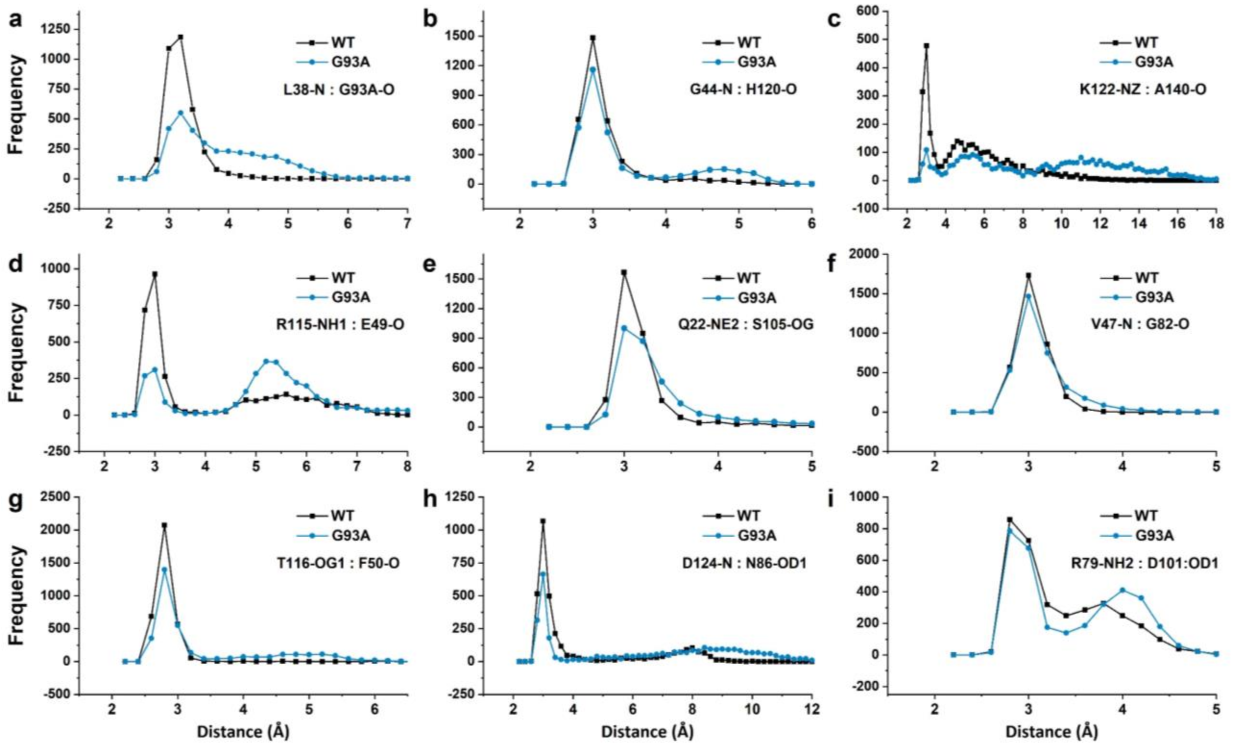

Supplementary Figure 6: Relevant distance distributions between atoms that form hydrogen bond interactions in the trajectories of WT SOD1 (black) and G93A SOD1 (blue), which includes the distance of L38(N) and G93A(O) (**a**), G44(N) and H120(O) (**b**), K122(NZ) and A140(O) (**c**), R115(NH1) and E49(O) (**d**), Q22(NE2) and S105(OG) (**e**), V47(N) and G82(O) (**f**), T116(OG1) and F50(O) (**g**), D124(N) and N86(OD1) (**h**), and R79(NH2) and D101(OD1) (**i**).

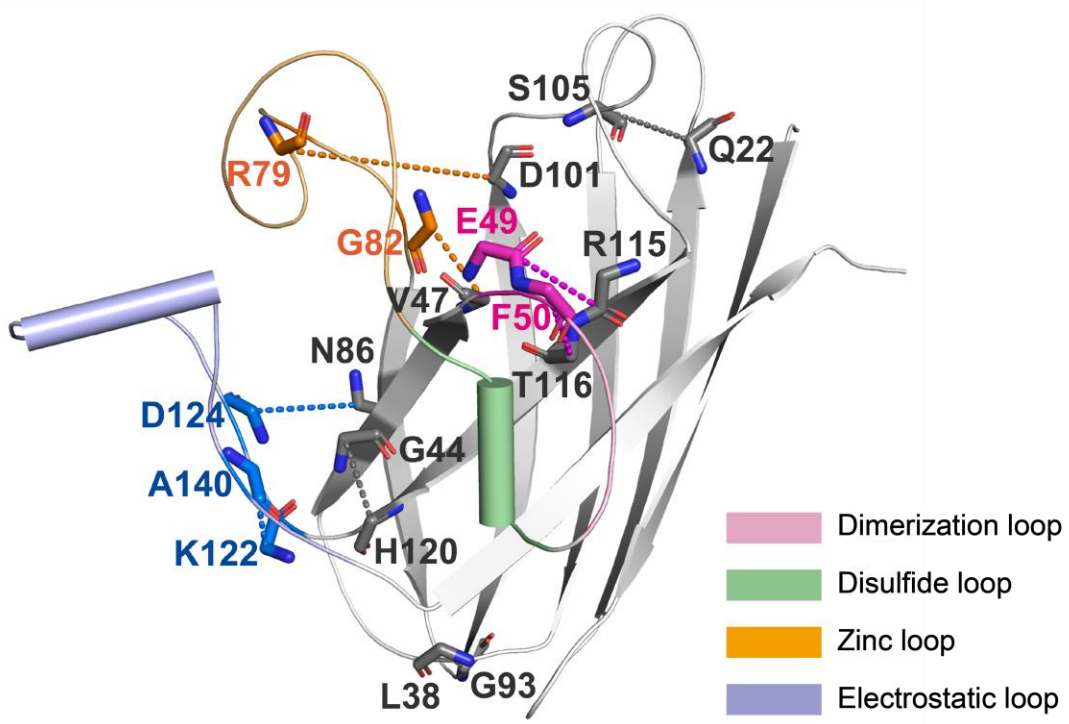

Supplementary Figure 7: SOD1 structure containing residues that form hydrogen bond interactions.

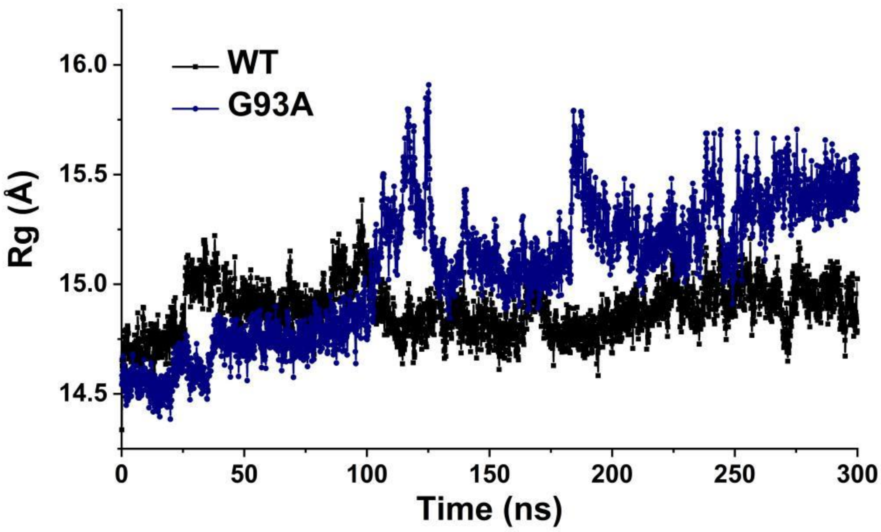

Supplementary Figure 8: Rg plot of WT SOD1 and G93A SOD1 systems.

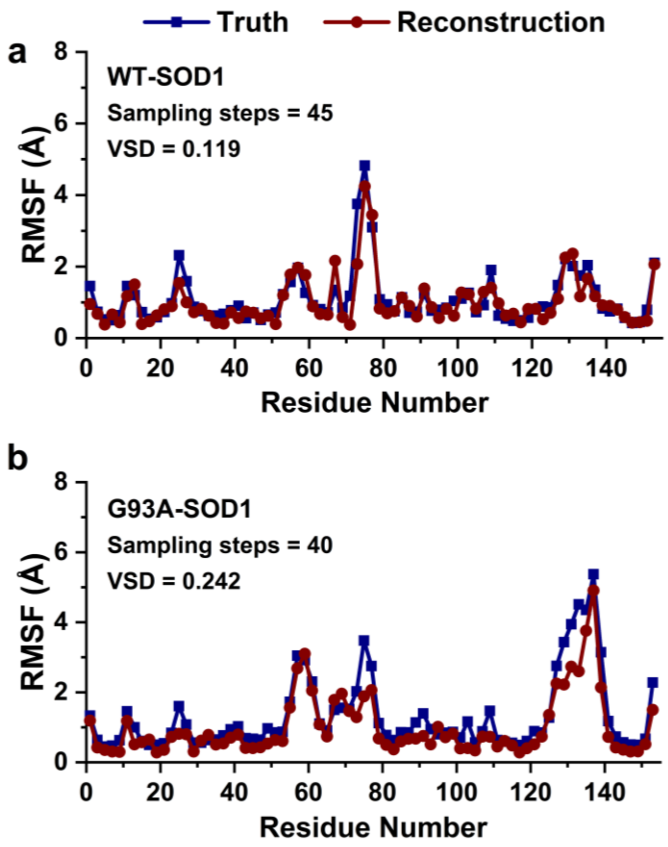

Supplementary Figure 9: Comparison of RMSF values between truth and reconstruction of trajectories for WT-SOD1 (**a**) and G93A-SOD1 (**b**). The VSD values are equal to 0.119 for WT-SOD1 and 0.242 for G93A-SOD1. The trajectories of WT and G93A-SOD1 were modeled using 45 and 40 sampling steps, respectively.

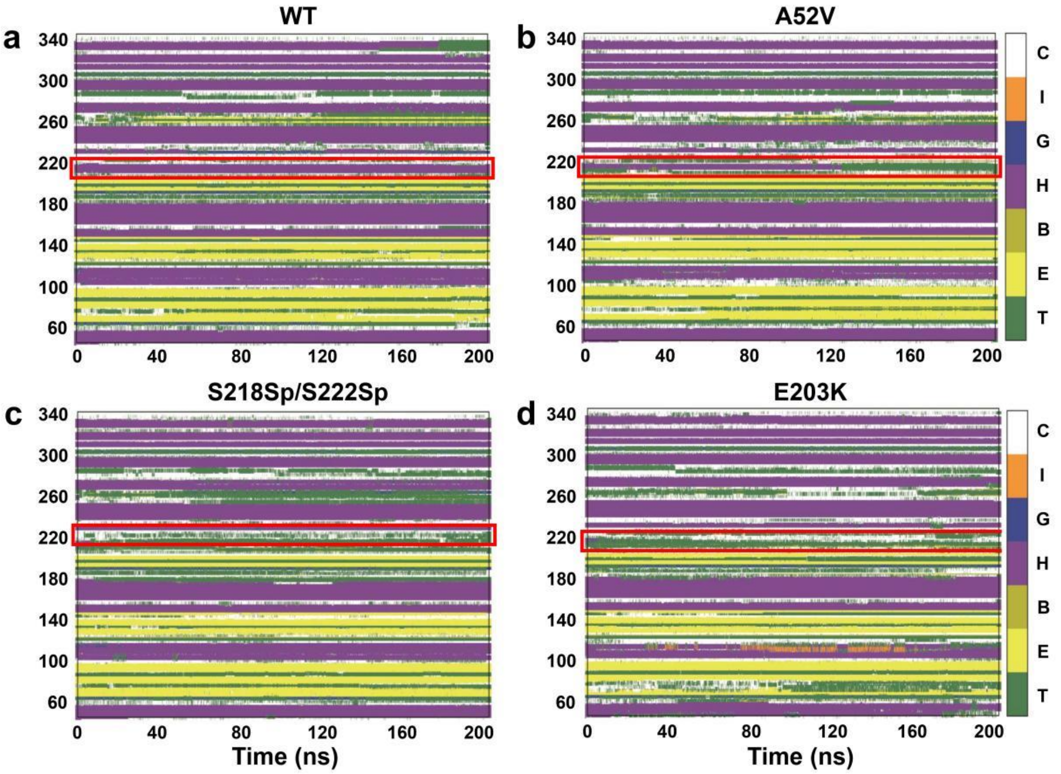

Supplementary Figure 10: Secondary structure analysis for WT (**a**), A52V (**b**), S218Sp/S222Sp (**c**), and E203K (**d**) of MEK1.

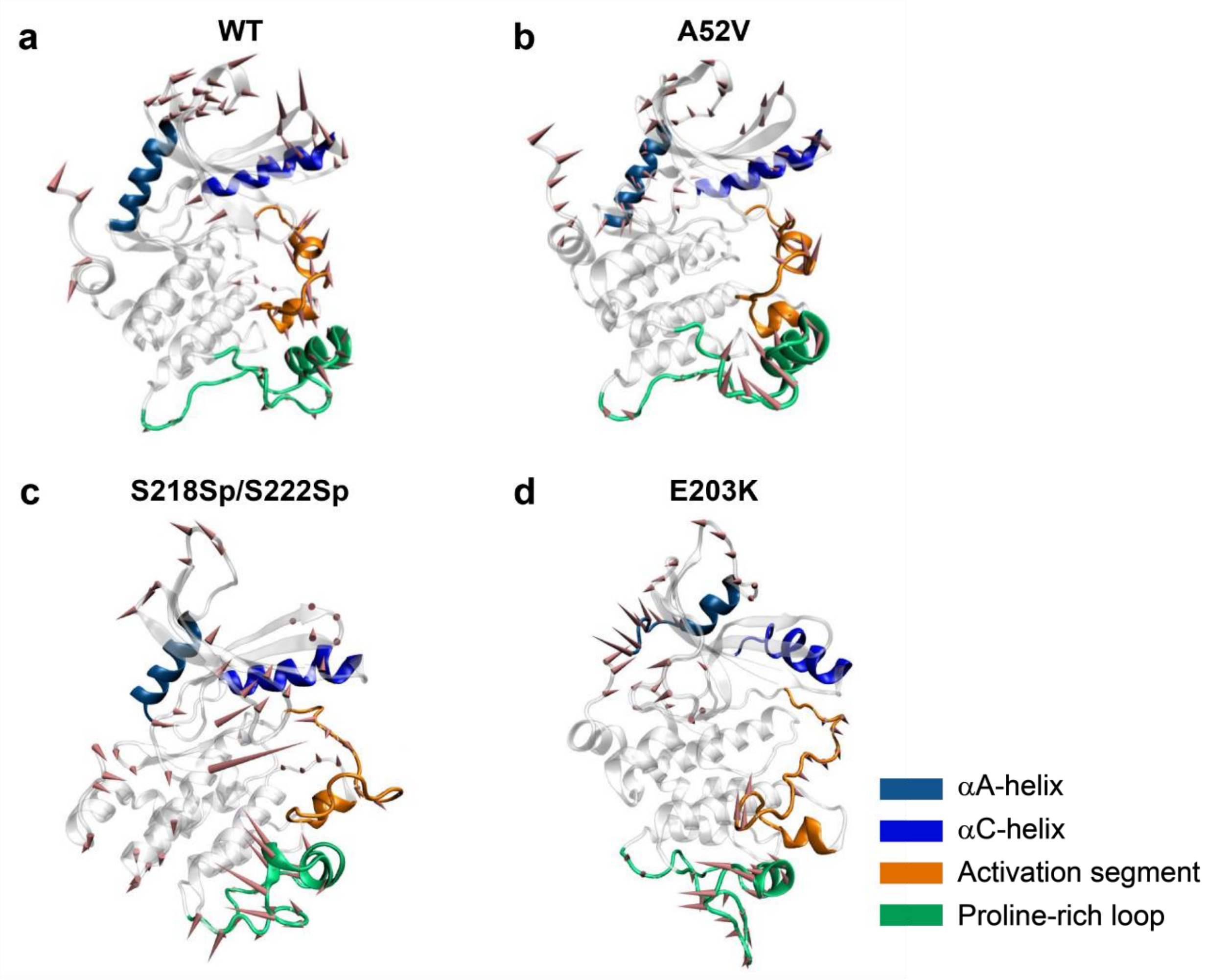

Supplementary Figure 11: Principal component analysis (PCA) for WT (**a**), A52V (**b**), S218Sp/S222Sp (**c**), and E203K (**d**) of MEK1. The directions shown in the graphics denote the motion mode of each protein.

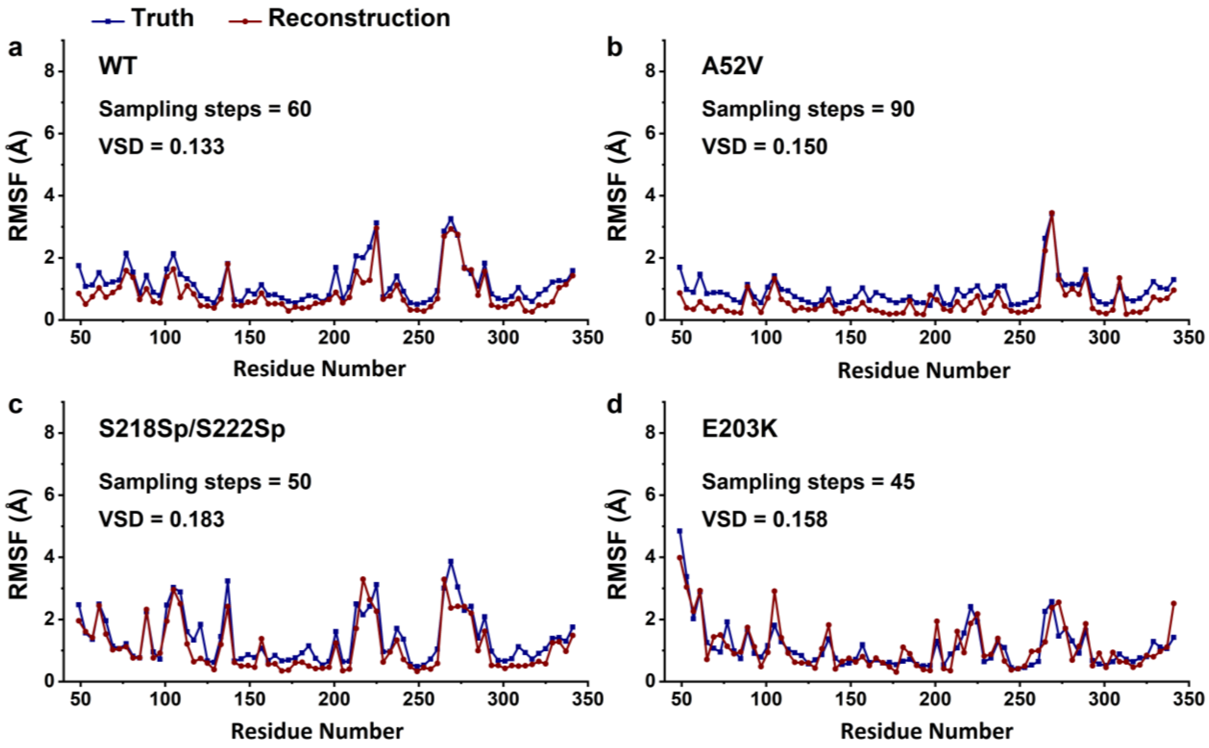

Supplementary Figure 12: Comparison of RMSF values between the simulation and the reconstruction of trajectories for WT (**a**), A52V (**b**), S218Sp/S222Sp (**c**), and E203K (**d**) of MEK1. The VSD values for WT, A52V, S218Sp/S222Sp, and E203K of MEK1 in various sampling steps are shown in the figure.

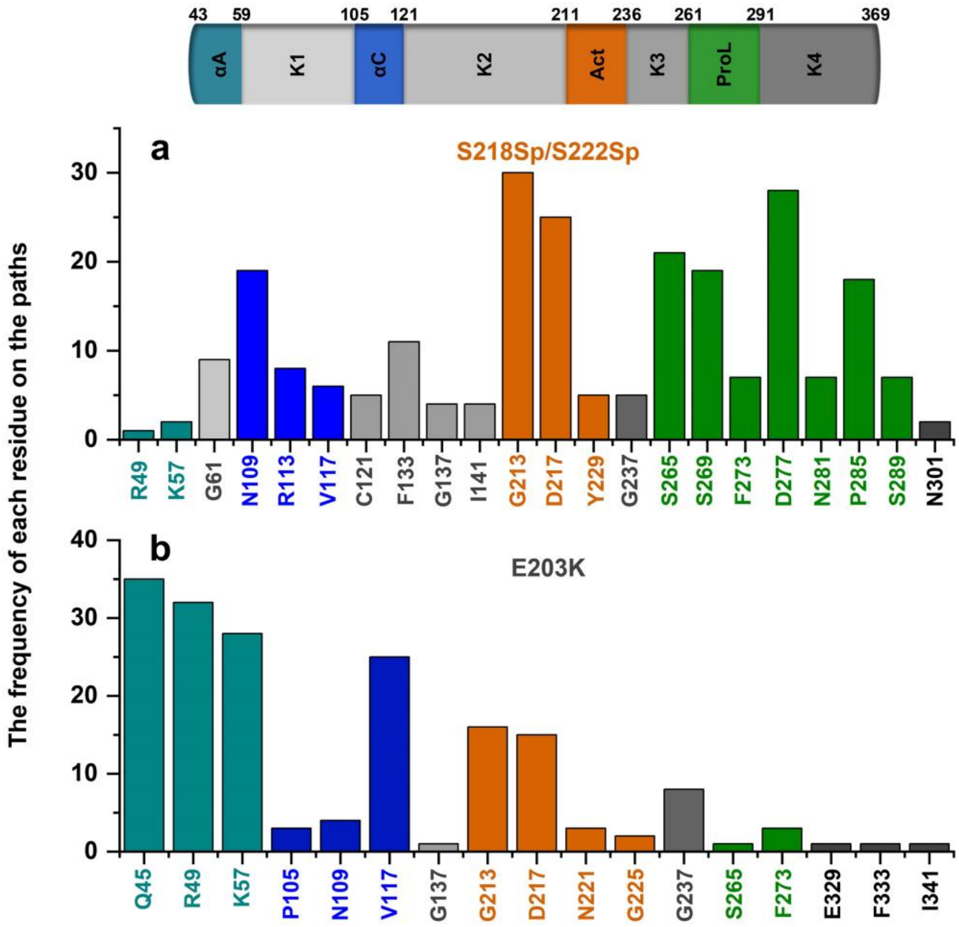

Supplementary Figure 13: Frequency of each residue on the shortest pathways for S218Sp/S222Sp (**a**) and E203K (**b**) of MEK1. The domains presented here are αA-helix (αA), core kinase domain 1-3 (K1-3), αC-helix (αC), activation segment (Act), and proline-rich loop (ProL).

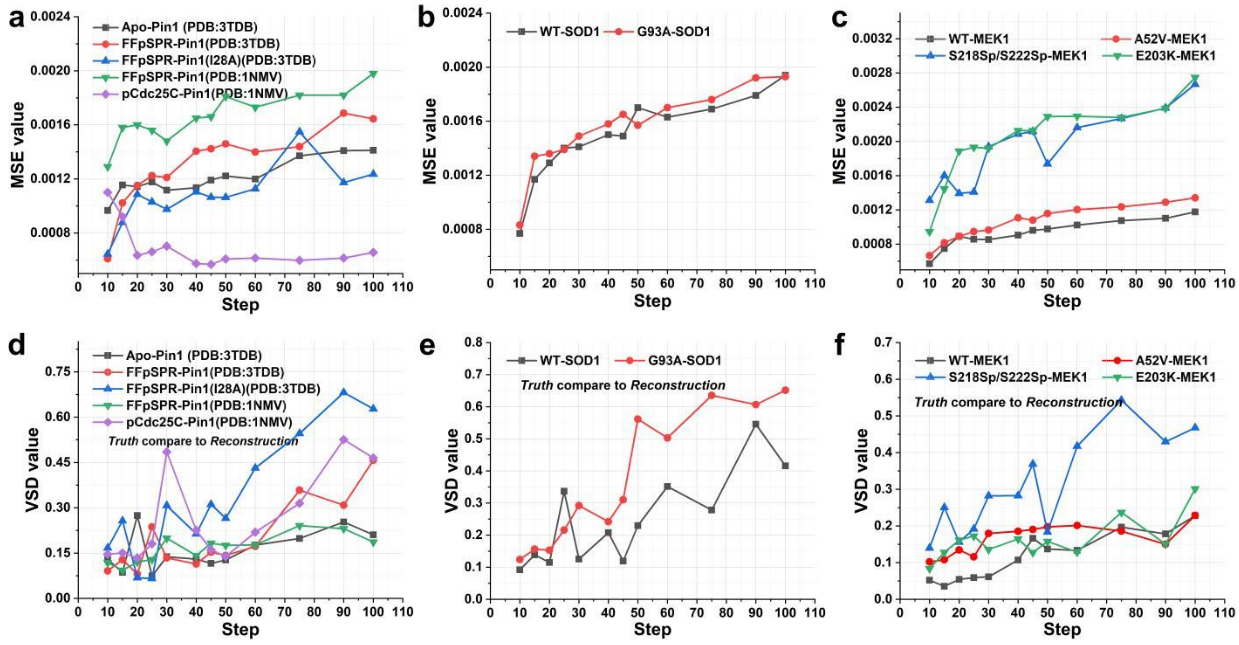

Supplementary Figure 14: Mean squared error (MSE) for the Pin1 (**a**), SOD1 (**b**), and MEK1 (**c**) systems. Value square deviation (VSD) of RMSF values of the ground truth and reconstructed trajectories for the Pin1 (**d**), SOD1 (**e**), and MEK1 (**f**) systems. We modeled the trajectories using a series of increased sampling steps. In general, the MSE and VSD values become larger as the sampling frequency increases.

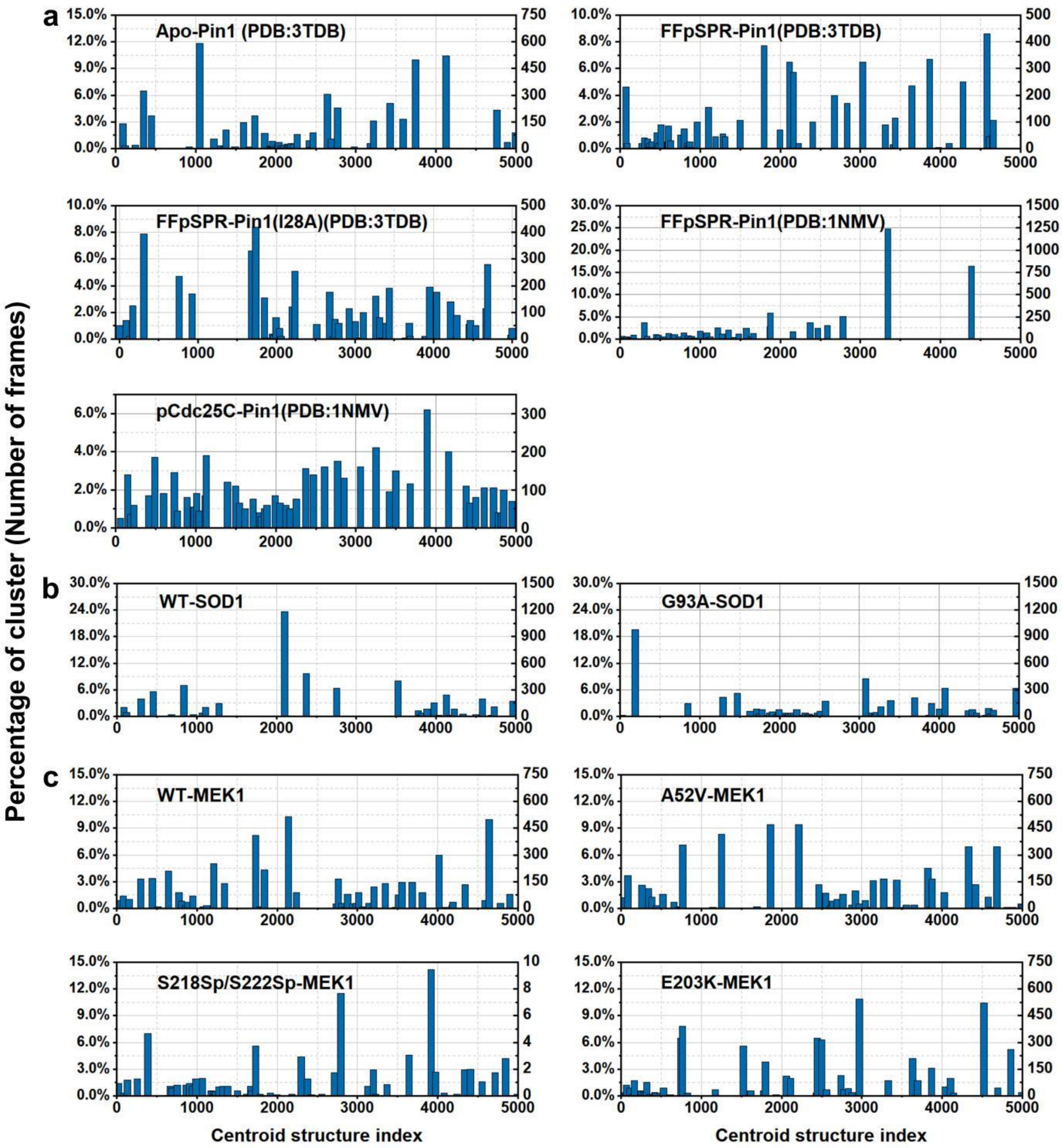

Supplementary Figure 15: Clustering results of Pin1 (**a**), SOD1 (**b**), and MEK1 (**c**) systems. The X-axis represents the index of the centroid structure in each cluster sorted by simulation time. The Y-axis represents the percentage of the total processed frames, i.e., the number of frames contained in each cluster.

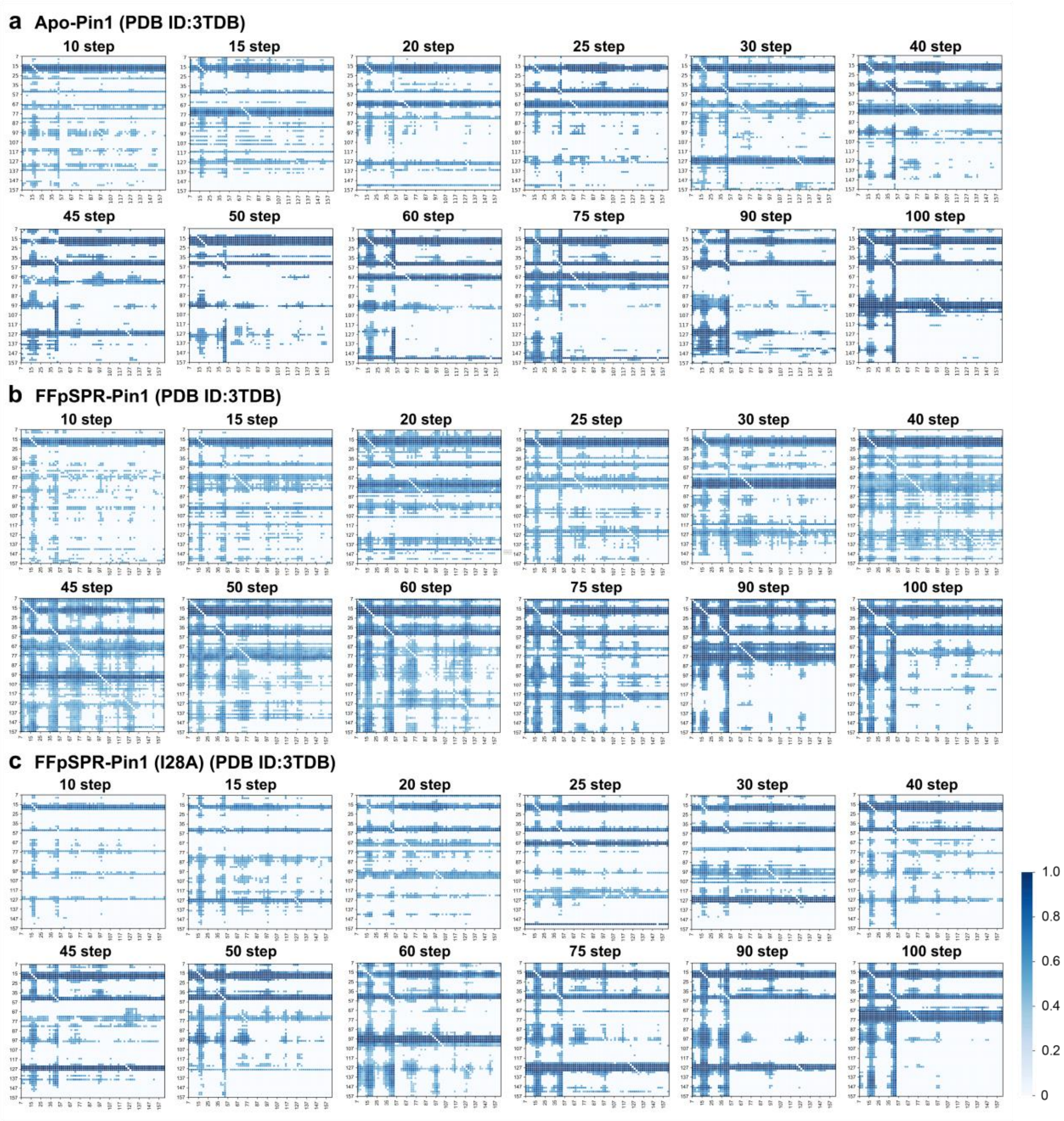

Supplementary Figure 16: The distribution of learned edges for the Apo-Pin1 (**a**), FFpSPR-bound Pin1 (**b**), and FFpSPR-bound Pin1 (I28A) (**c**), obtained from the modeling using different sampling steps.

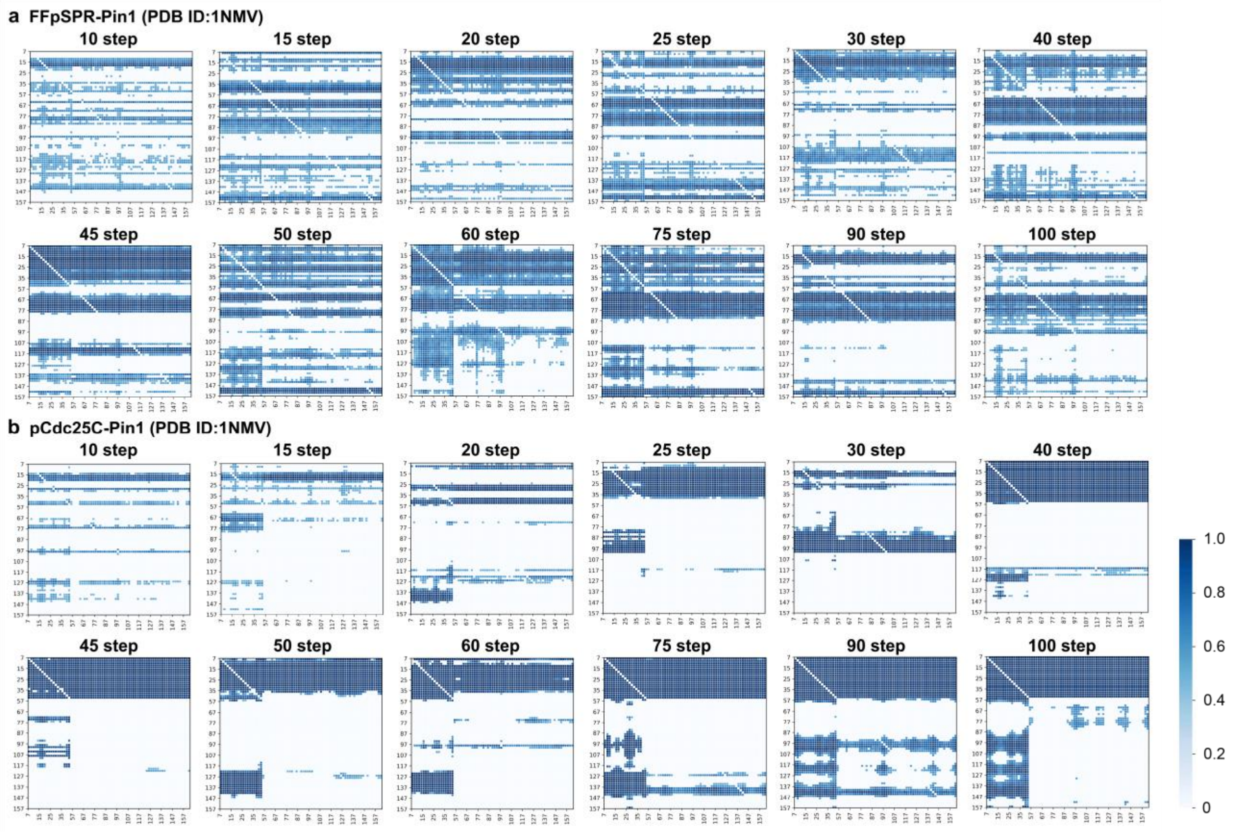

Supplementary Figure 17: The distribution of learned edges for the FFpSPR-bound Pin1 (**a**) and pCdc25C-bound Pin1 (**b**) with two domains-separated, obtained from the modeling using different sampling steps.

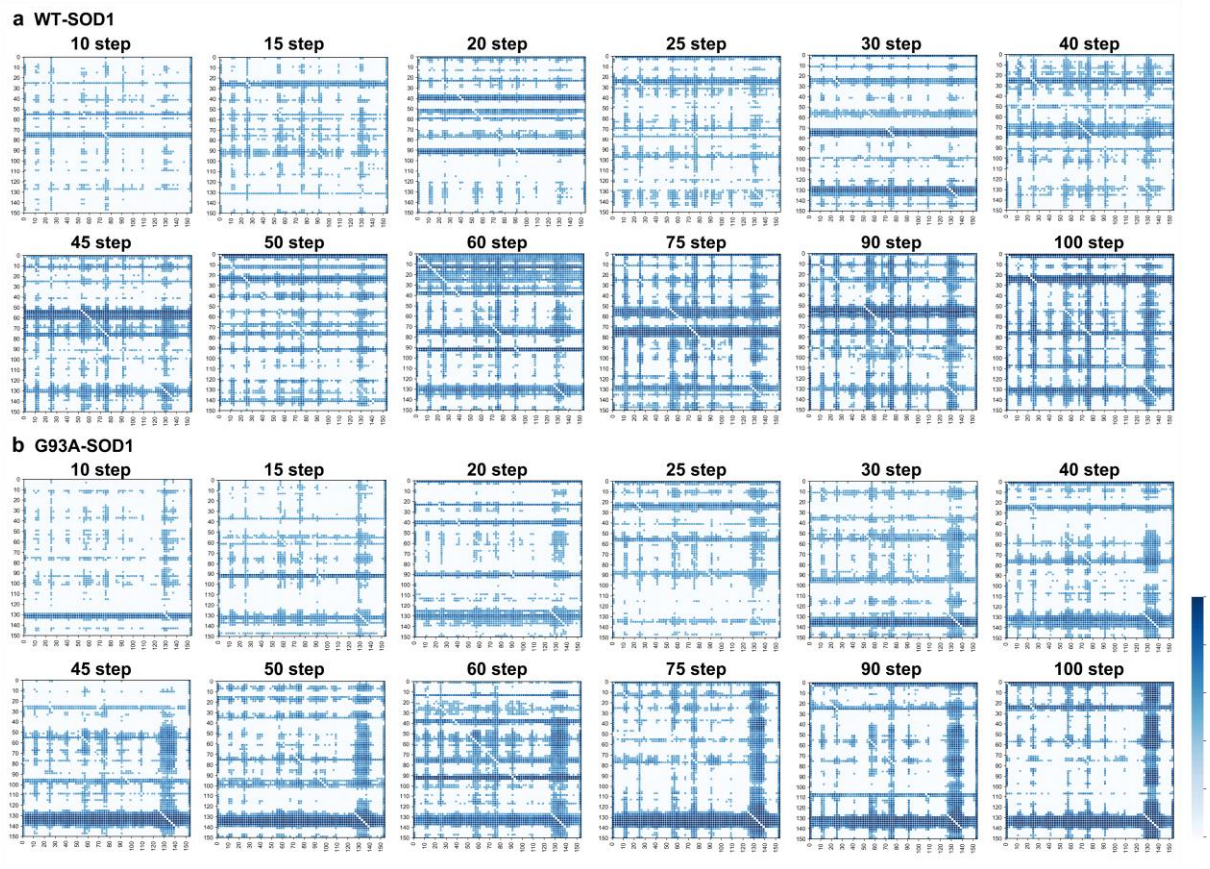

Supplementary Figure 18: The distribution of learned edges for the WT-SOD1 (**a**) and G93A-SOD1 (**b**), obtained from the modeling using different sampling steps.

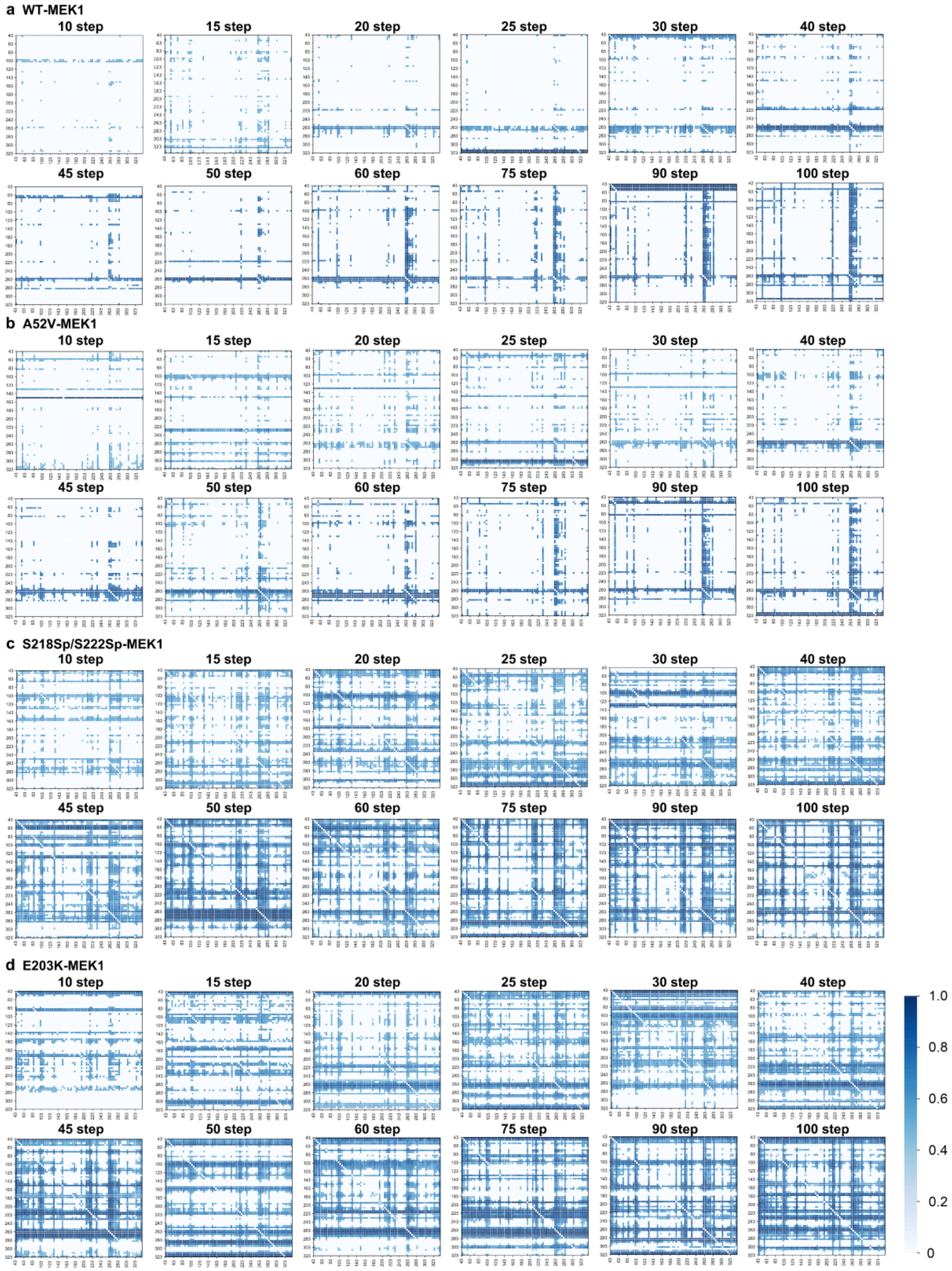

Supplementary Figure 19: The distribution of learned edges for the WT (**a**), A52V (**b**), S218Sp/S222Sp (**c**), and E203K (**d**) MEK1 systems, obtained from the modeling using different sampling steps.

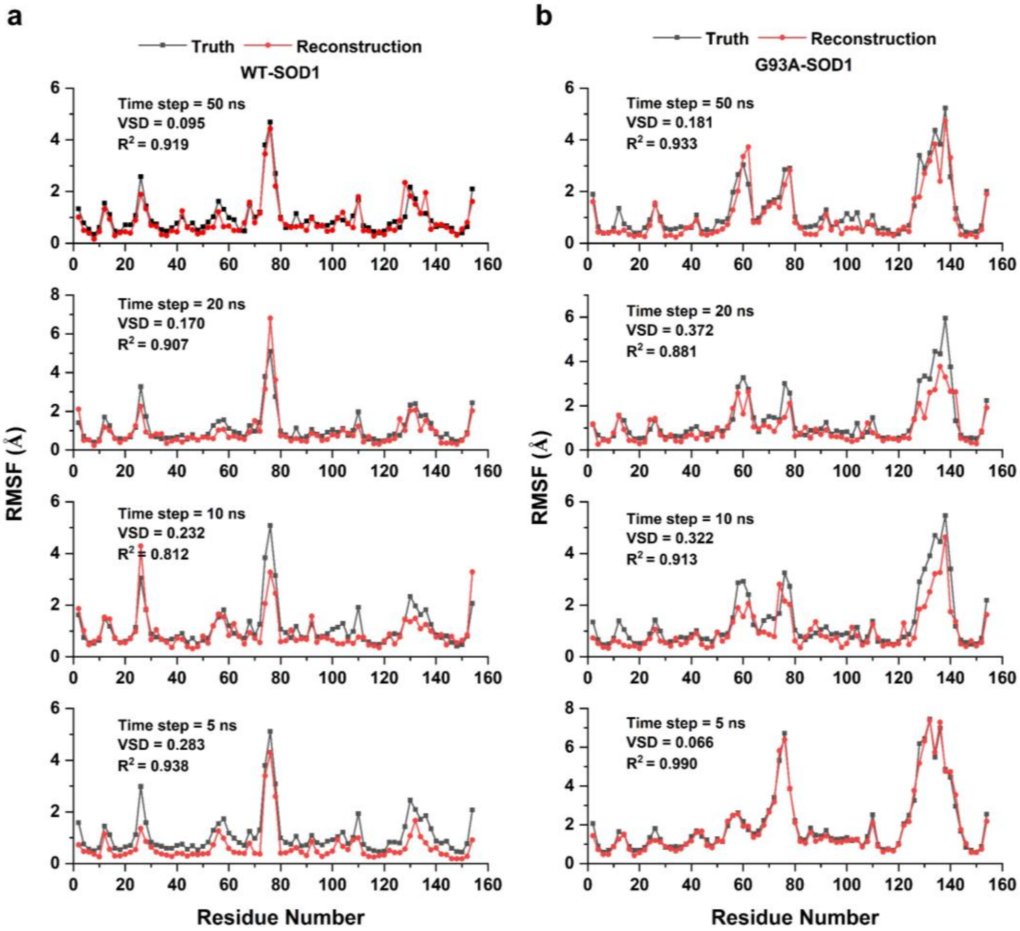

Supplementary Figure 20: Comparison of RMSF values between the simulation and the reconstruction of trajectories for WT-SOD1 (**a**) and G93A-SOD1 (**b**). The trajectories of WT and G93A-SOD1 were modeled using time intervals of 50, 20, 10, and 5 ns (sampling steps of 10, 25, 50, and 100, respectively). The total simulation time is 500 ns.

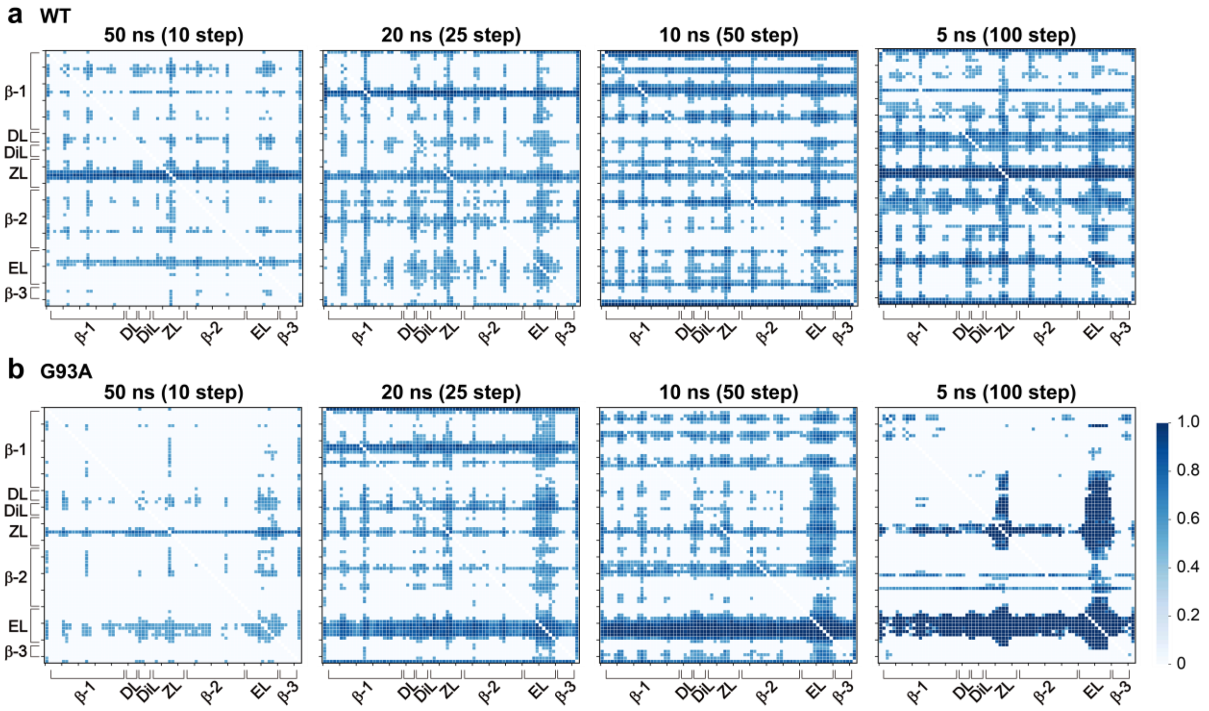

Supplementary Figure 21: The distribution of learned edges for WT-SOD1 (**a**) and G93A-SOD1 (**b**), obtained from the modeling using time intervals of 50, 20, 10, and 5 ns (sampling step with 10, 25, 50, and 100, respectively). The total simulation time is 500 ns. The main domains of SOD1 are consist of the β-barrel (β1-3), Dimerization loop (DL), Disulfide loop (DiL), Zinc loop (ZL), and Electrostatic loop (EL).

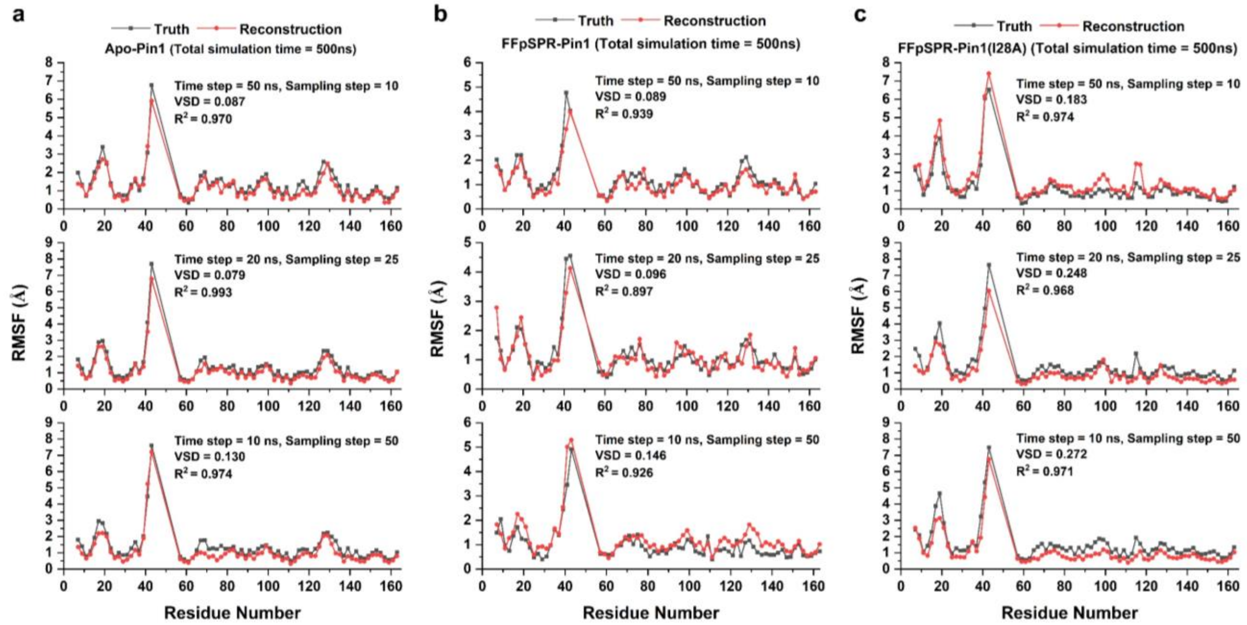

Supplementary Figure 22: Comparison of RMSF values between the simulation and the reconstruction of trajectories for apo Pin1 (**a**), FFpSPR-bound Pin1 (**b**), FFpSPR-bound Pin1 (I28A) (**c**). The trajectories of the Pin1 complexes were modeled using time intervals of 50, 20, and 10 ns (sampling step with 10, 25, and 50, respectively). The total simulation time is 500 ns.

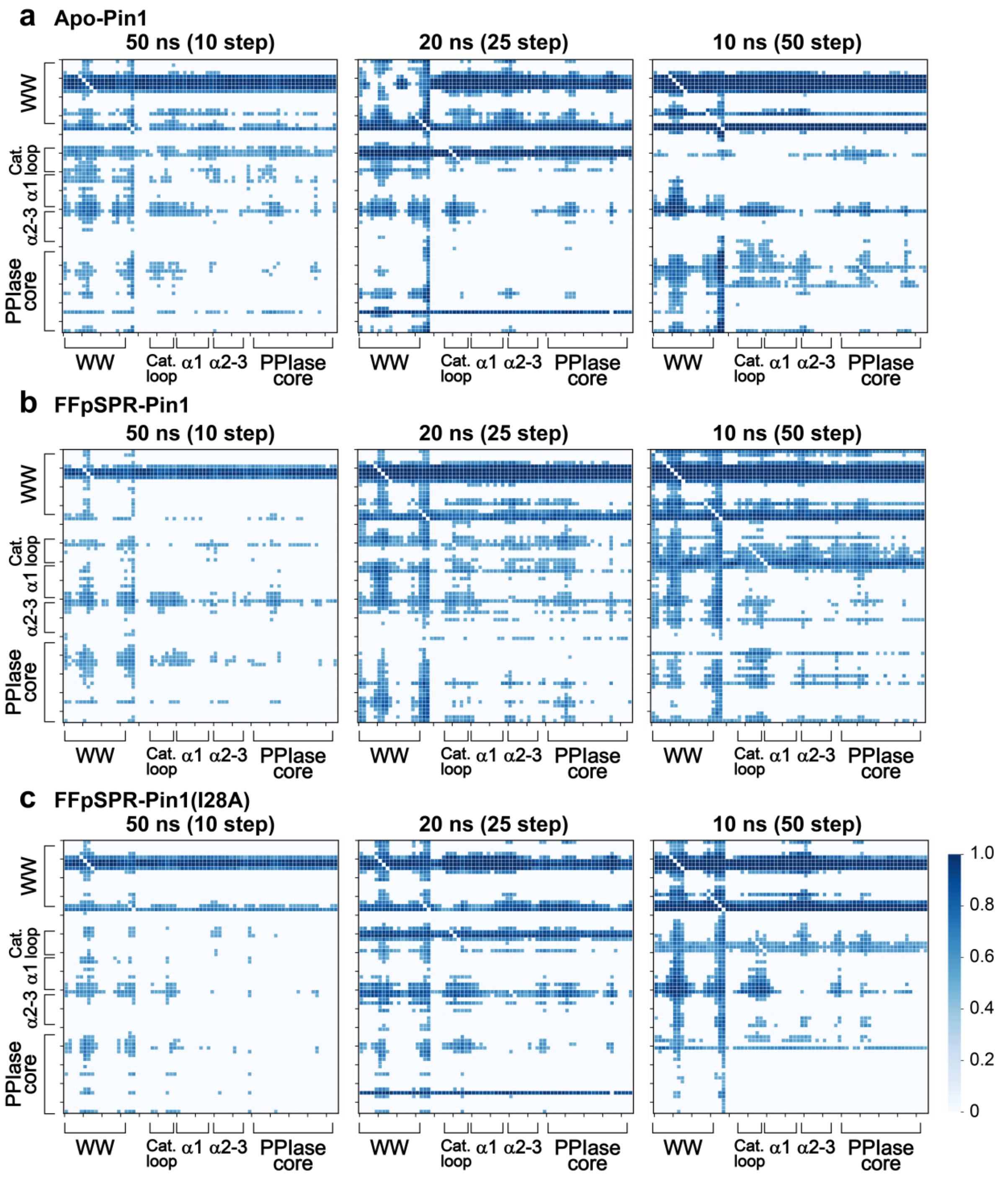

Supplementary Figure 23: The distribution of learned edges for the Apo-Pin1 (**a**), FFpSPR-bound Pin1 (**b**), and FFpSPR-bound Pin1 (I28A) (**c**), obtained from the modeling using time intervals of 50, 20, and 10 ns (sampling step with 10, 25, and 50, respectively). The total simulation time is 500 ns. The domains presented here are WW domain (WW), catalytic loop (Cat. loop), α1-helix (α1), α2-3 helices (α2-3), and PPIase core.

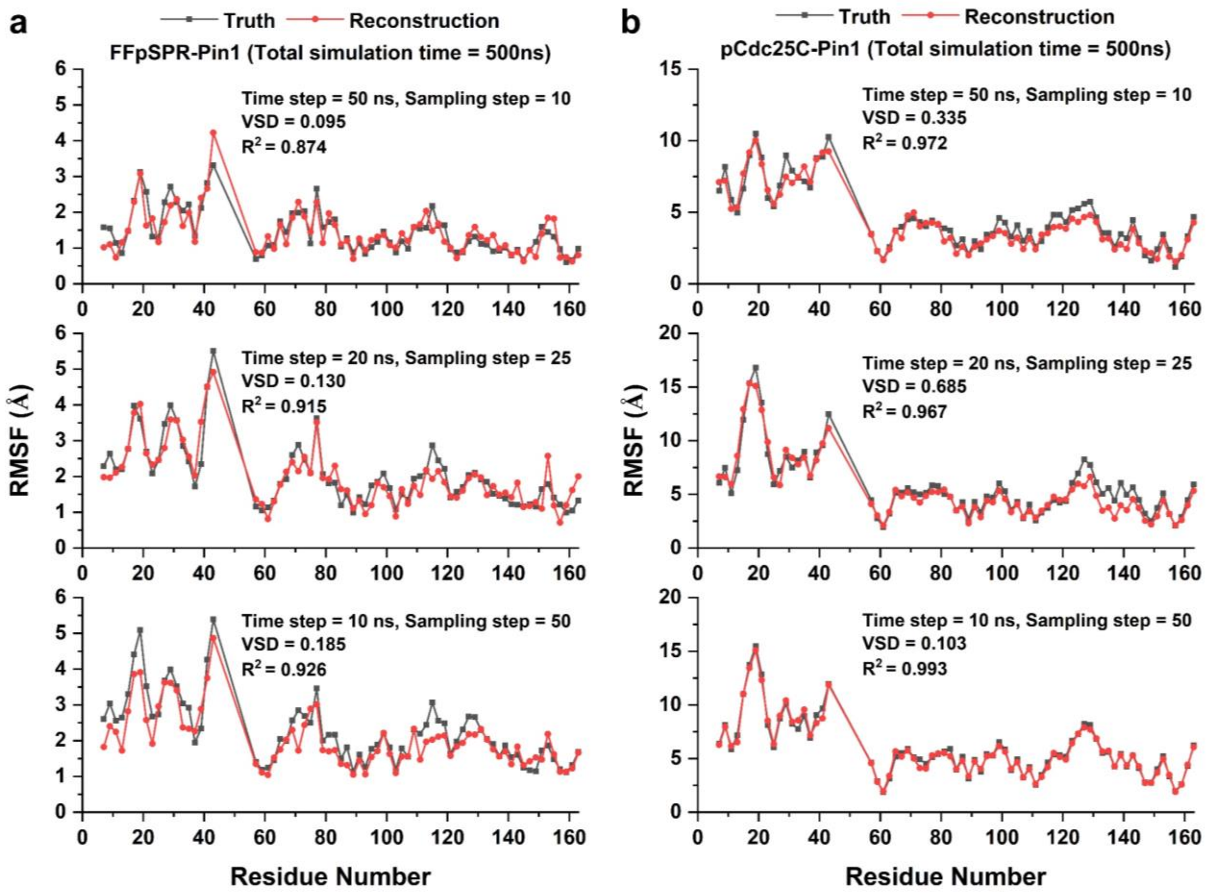

Supplementary Figure 24: Comparison of RMSF values between the simulation and the reconstruction of trajectories for FFpSPR-bound Pin1 with two domains-separated (**a**), and pCdc25C-bound Pin1 with two domains-separated (**b**). The trajectories of Pin1 complexes were modeled using time intervals of 50, 20, and 10 ns (sampling step with 10, 25, and 50, respectively). The total simulation time is 500 ns.

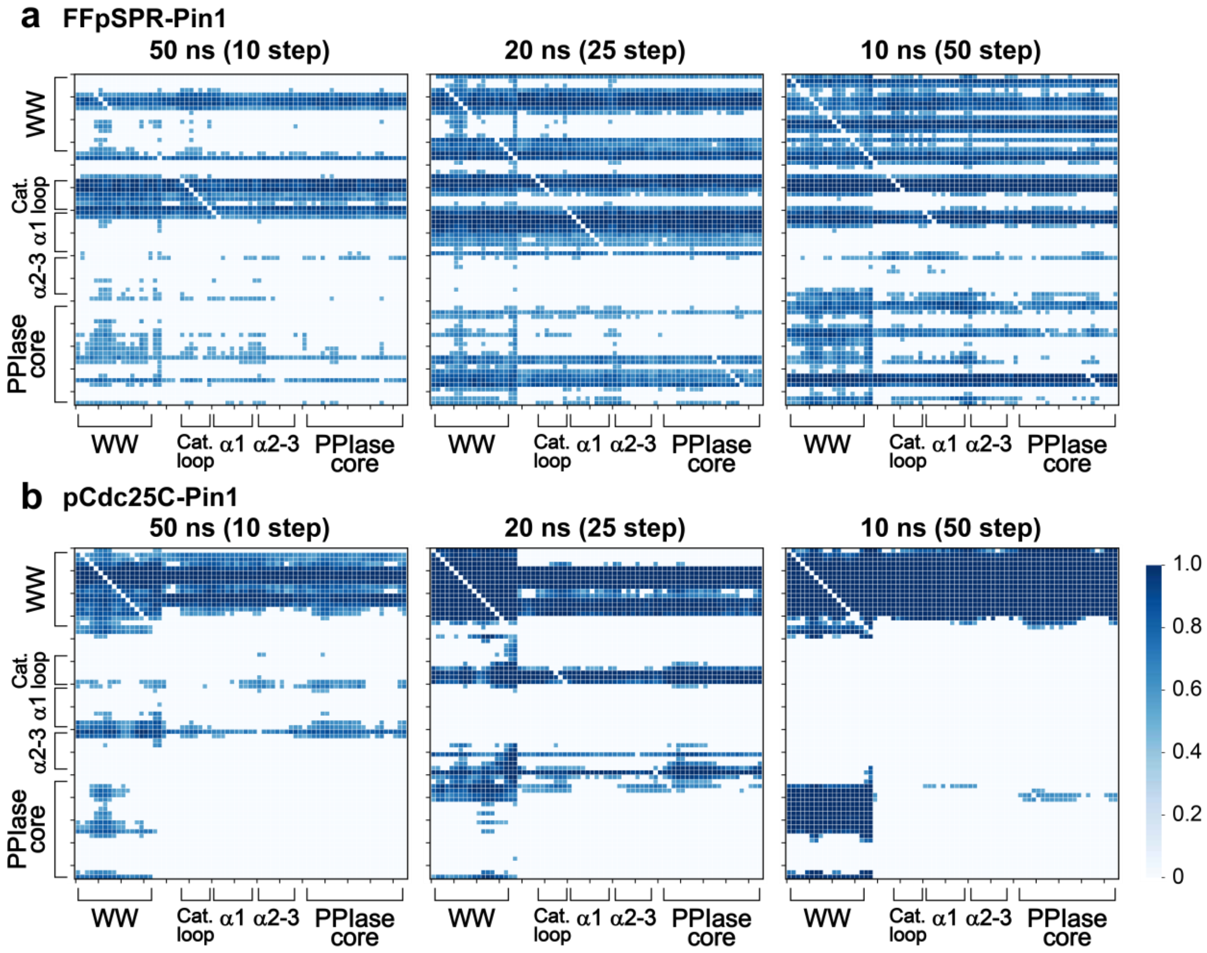

Supplementary Figure 25: The distribution of learned edges for FFpSPR-bound Pin1 with two domains-separated (**a**), and pCdc25C-bound Pin1 with two domains-separated (**b**), obtained from the modeling using time intervals of 50, 20, and 10 ns (sampling step with 10, 25, and 50, respectively). The total simulation time is 500 ns. The domains presented here are WW domain (WW), catalytic loop (Cat. loop), α1-helix (α1), α2-3 helices (α2-3), and PPIase core.

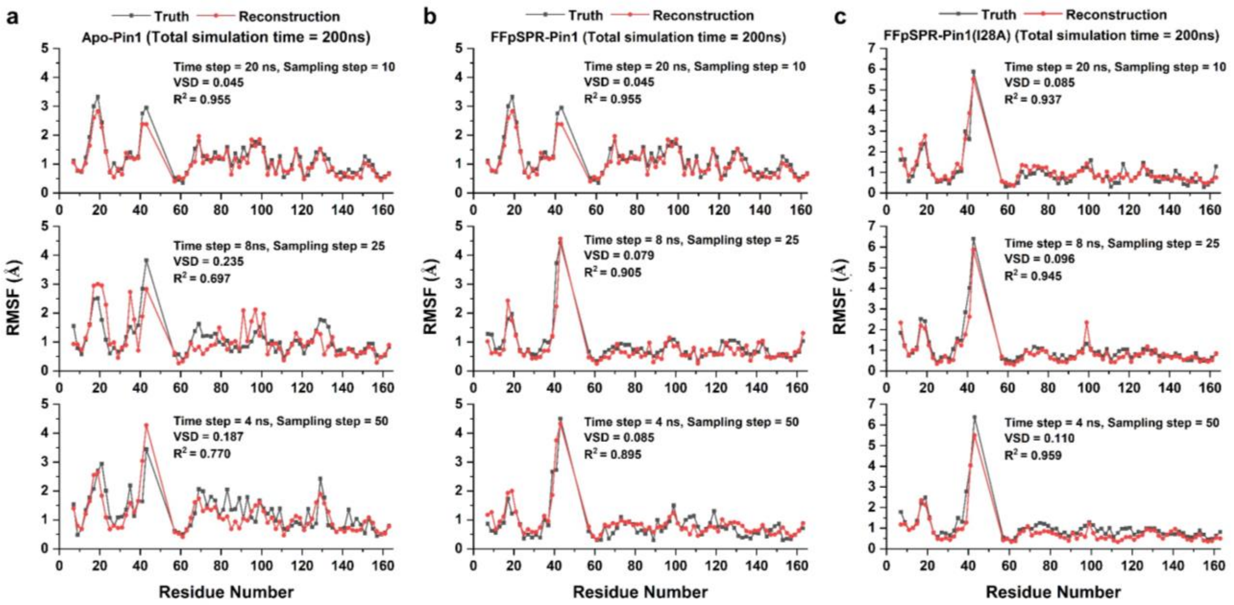

Supplementary Figure 26: Comparison of RMSF values between the simulation and the reconstruction of trajectories for apo Pin1 (**a**), FFpSPR-bound Pin1 (**b**), FFpSPR-bound Pin1 (I28A) (**c**). The trajectories of Pin1 complexes were modeled using time intervals of 20, 8, and 4 ns (sampling step with 10, 25, and 50, respectively). The total simulation time is 200 ns.

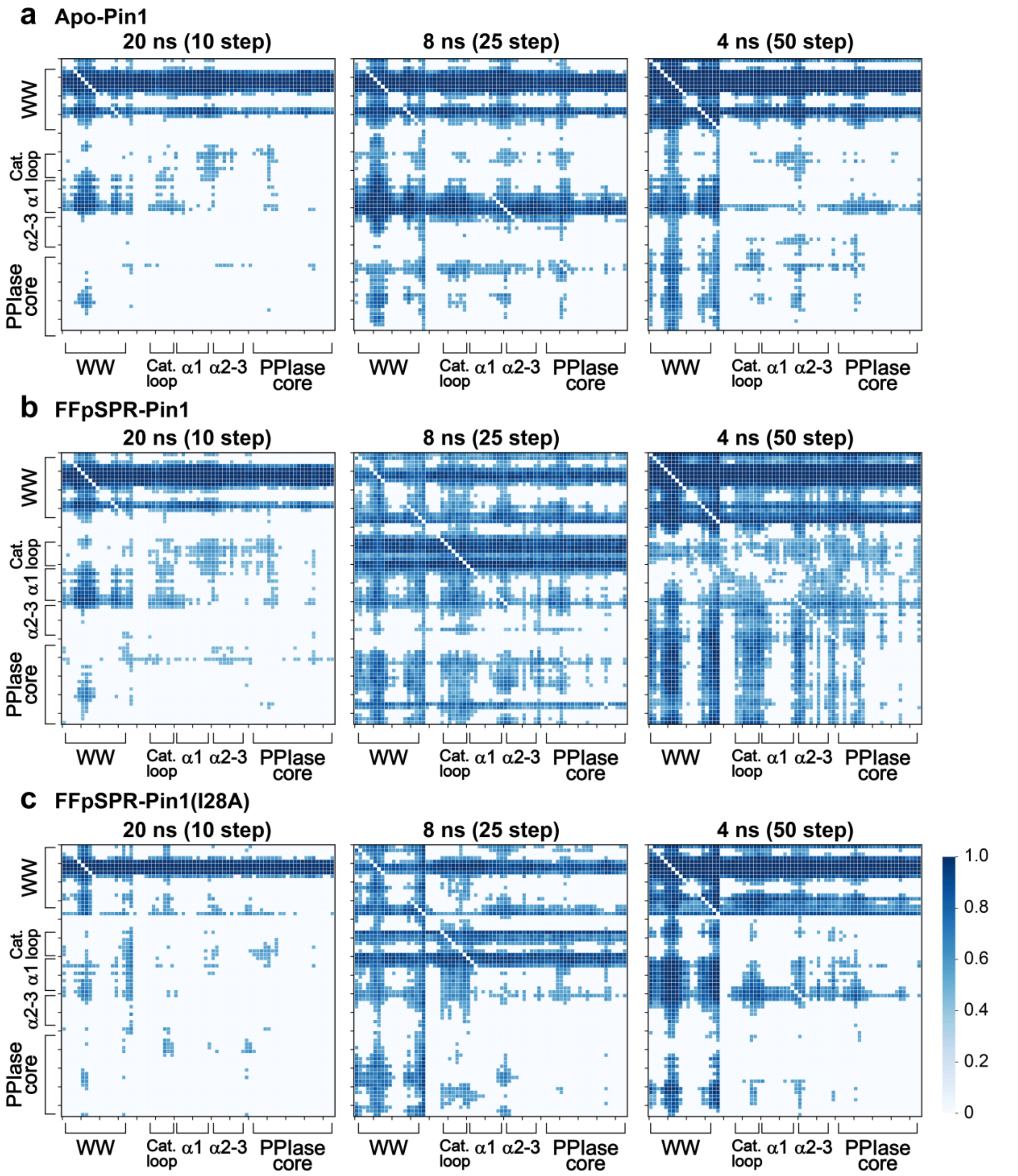

Supplementary Figure 27: The distribution of learned edges for the Apo-Pin1 (**a**), FFpSPR-bound Pin1 (**b**), and FFpSPR-bound Pin1 (I28A) (**c**), obtained from the modeling using time intervals of 20, 8, and 4 ns (sampling step with 10, 25, and 50, respectively). The total simulation time is 200 ns. The domains presented here are WW domain (WW), catalytic loop (Cat. loop), α1-helix (α1), α2-3 helices (α2-3), and PPIase core.

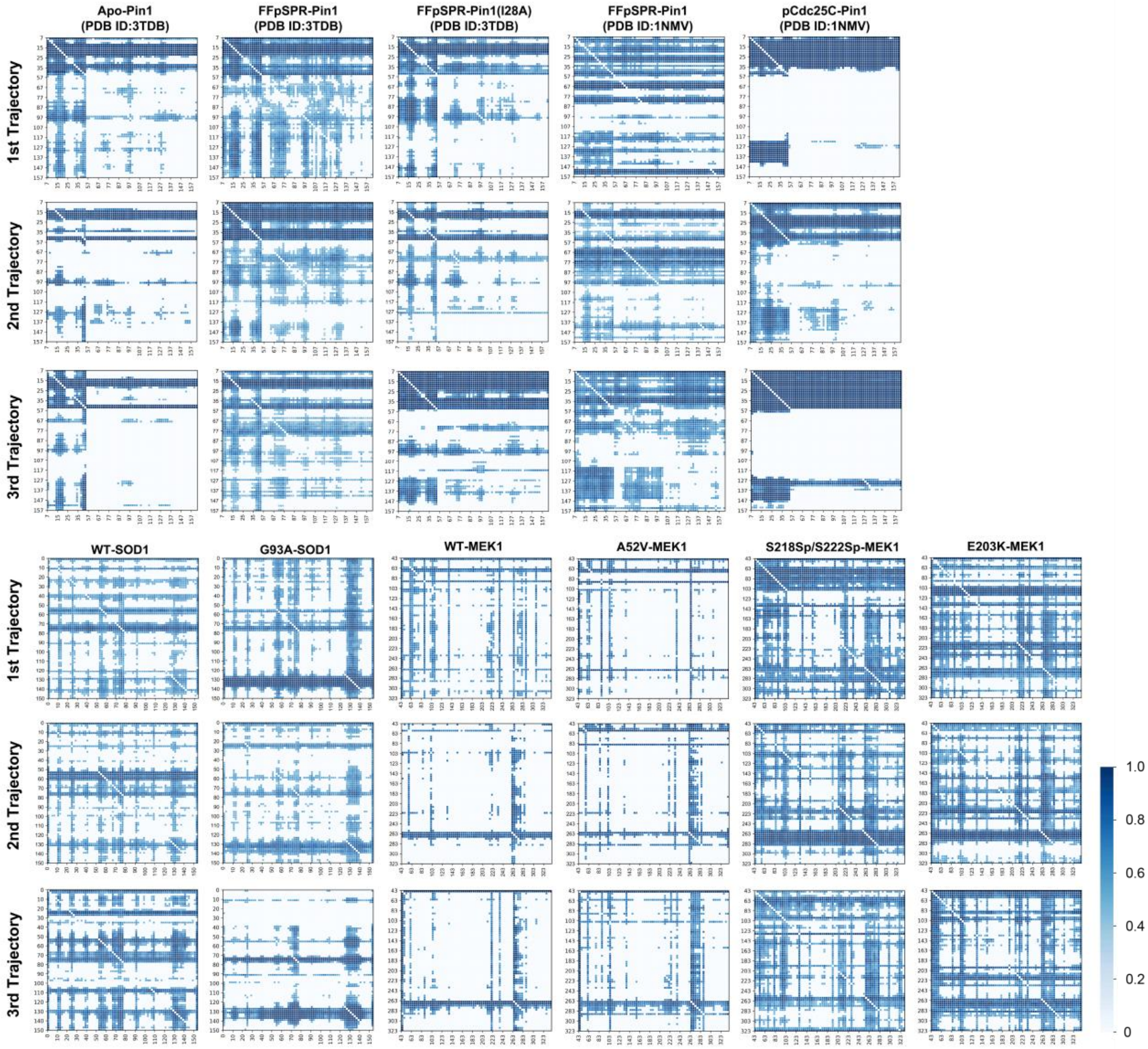

Supplementary Figure 28: The distribution of learned edges for the Pin1, SOD1, and MEK1 systems. The simulations for three case studies were repeated two additional times to validate the power and accuracy of our approach.

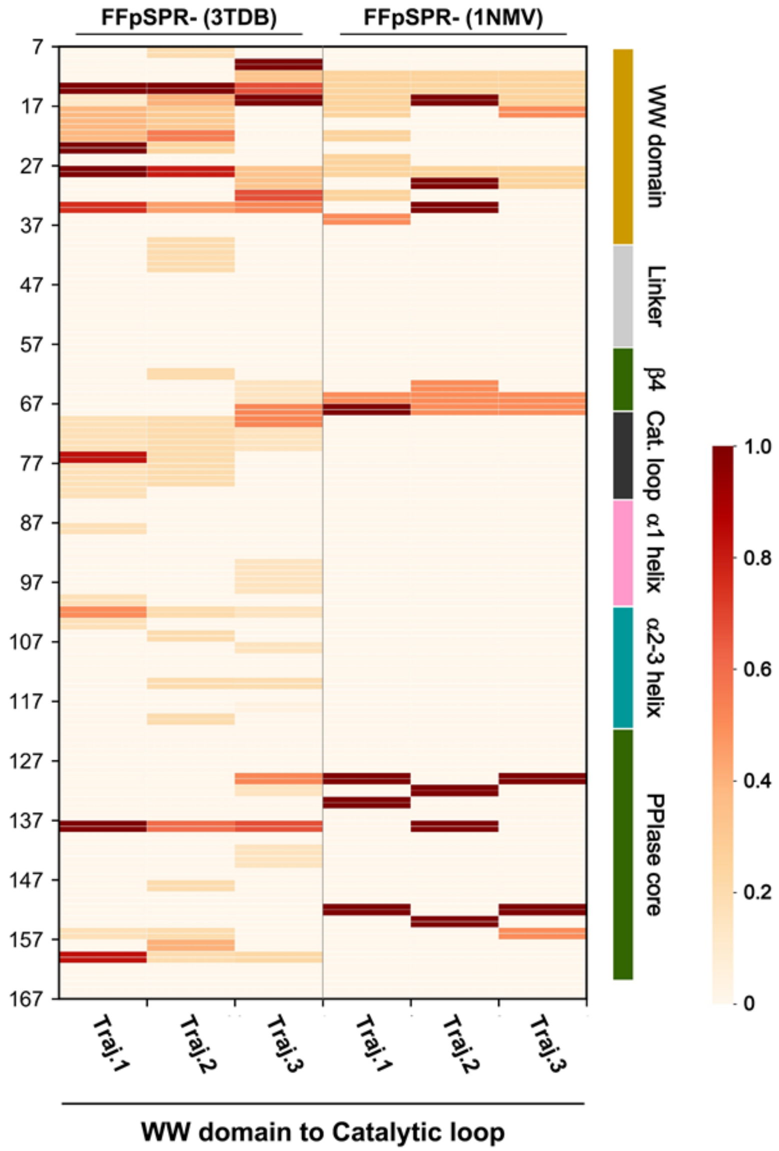

Supplementary Figure 29: The node centralities in the allosteric pathways between the WW domain and the catalytic loop for three repeated trajectories of FFpSPR-Pin1 complexes. The color scale bar (from 1.0->0.0) represents the decrease in the importance of a residue measured by normalized node centrality (i.e., the fraction of suboptimal paths going through the node or residue). The main domains of Pin1 are consist of the WW domain (WW), β4-sheet (β4), catalytic loop (Cat. loop), α1-helix (α1), α2-3 helices (α2-3), and PPIase core.

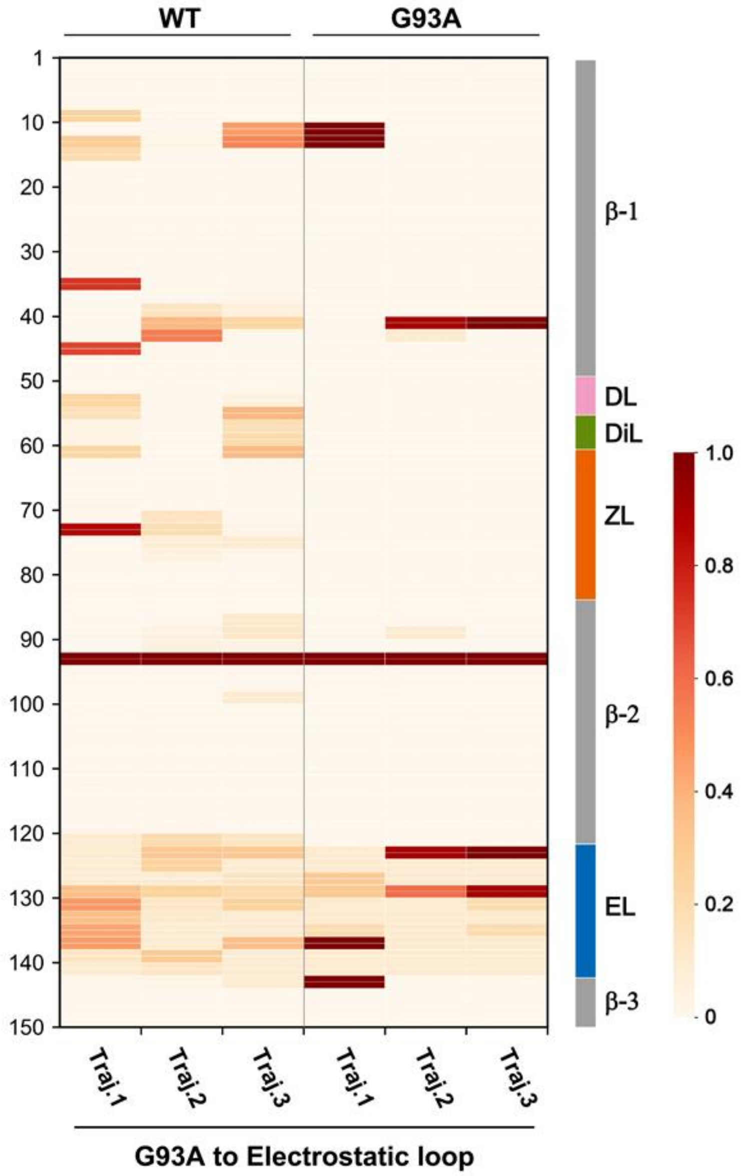

Supplementary Figure 30: The node centralities in the allosteric pathways between residue G93/A93 and the electrostatic loop for three repeated trajectories of WT- and G93A-SOD1 complexes. The main domains of SOD1 are consist of the β-barrel (β1-3), Dimerization loop (DL), Disulfide loop (DiL), Zinc loop (ZL), and Electrostatic loop (EL).

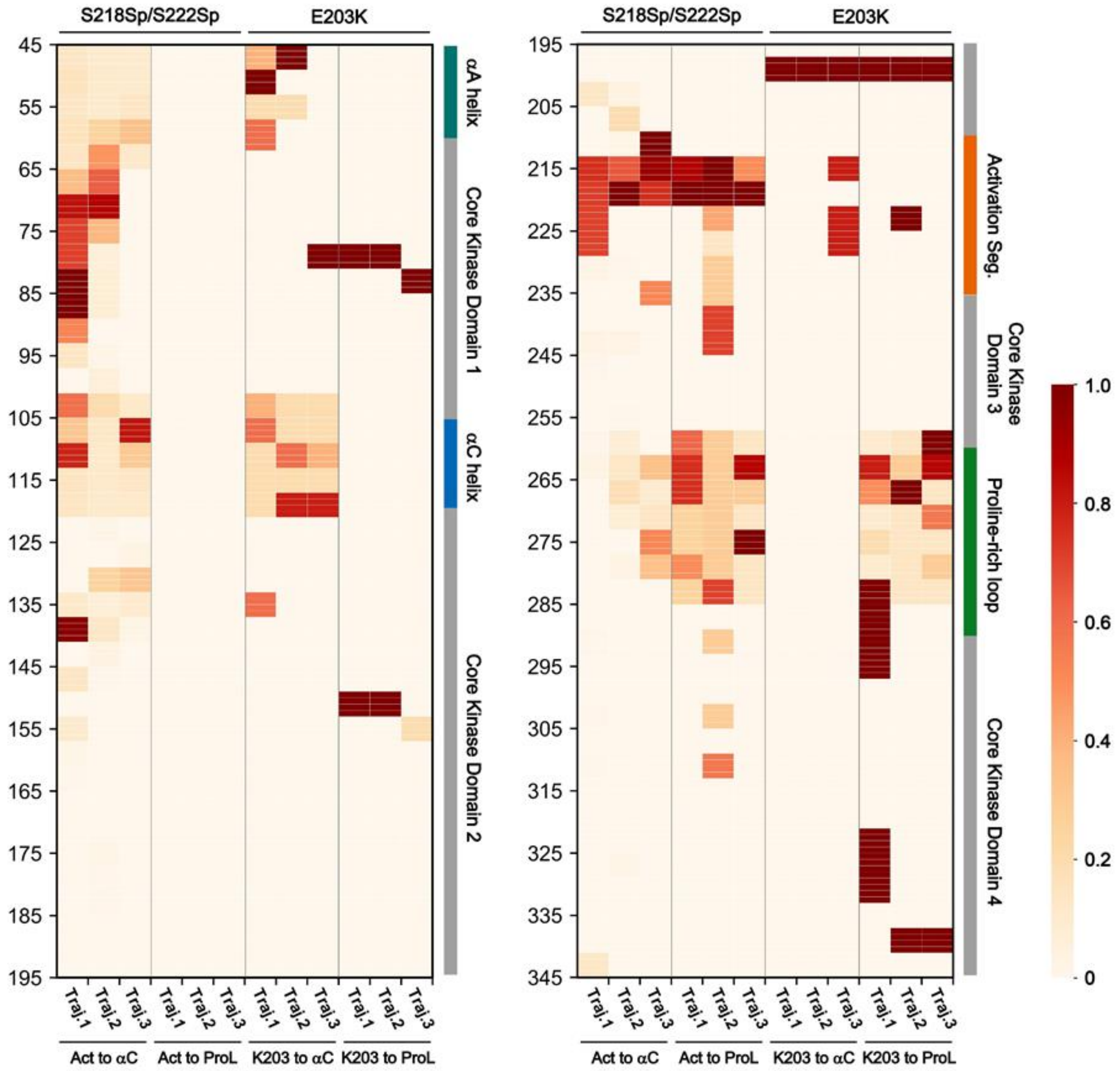

Supplementary Figure 31: The node centralities in the allosteric pathways between the activation segment and the αC-helix/proline-rich loop for three repeated trajectories of S218Sp/S222Sp- and E203K-MEK1 complexes.

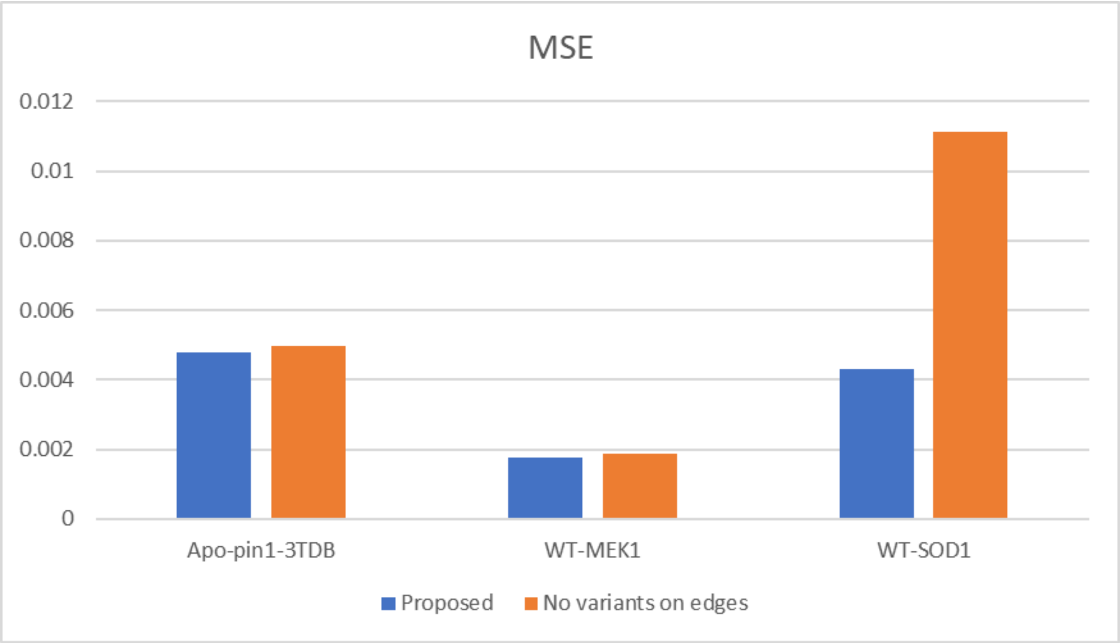

Supplementary Figure 32: Comparison of mean squared error (MSE) values between the proposed model and the model without latent variables on edges.

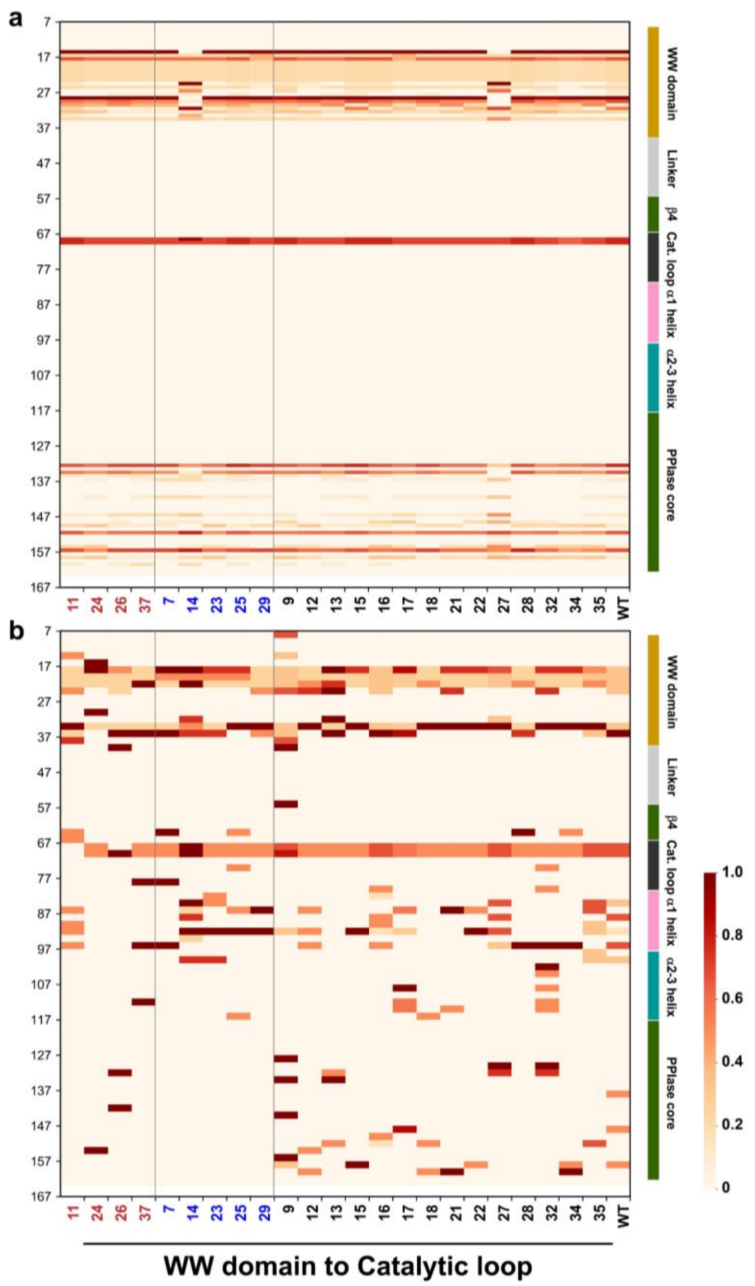

Supplementary Figure 33: The node centralities in the allosteric pathways between the WW domain and the catalytic loop for the WT and 23 Ala-mutants of unbound Pin1, which are calculated based on the covariance matrices obtained from constraint network analysis (CNA) (**a**) and the NRI model (**b**). Mutations that destabilize more than 3 kcal/mol are shown as red numbers, more than 1 kcal/mol and less than 3 kcal/mol are shown as blue numbers, and less than 1 kcal/mol are shown as black numbers. The color scale bar (from 1.0->0.0) represents the decrease in the importance of a residue measured by normalized node centrality (i.e., the fraction of suboptimal paths going through the node or residue). The main domains of Pin1 are consist of the WW domain (WW), β4-sheet (β4), catalytic loop (Cat. loop), α1-helix (α1), α2-3 helices (α2-3), and PPIase core.

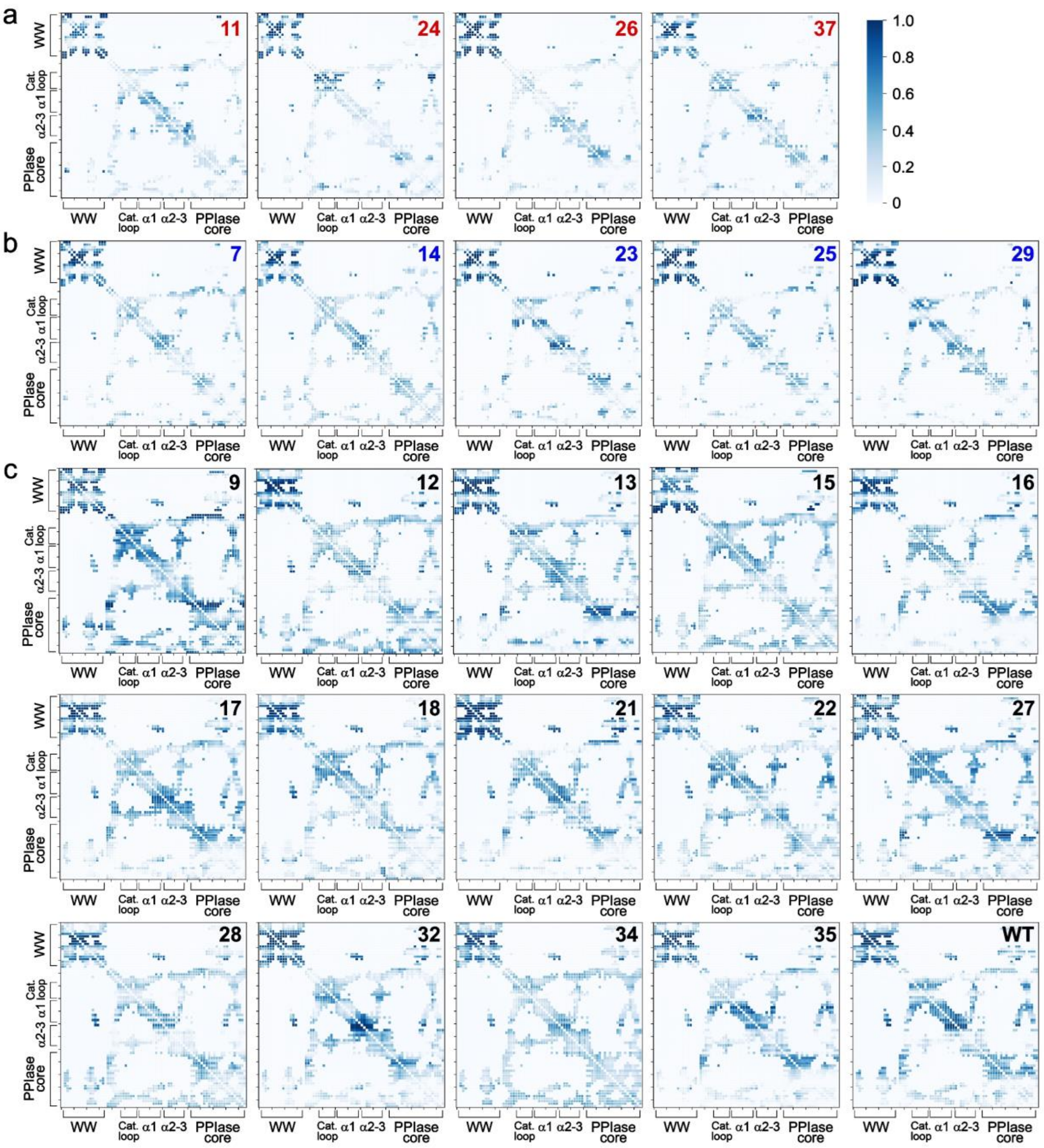

Supplementary Figure 34: Residue-residue interaction maps obtained by the NRI model for the WT and 23 Ala-mutants of unbound Pin1. Mutations that destabilize more than 3 kcal/mol are shown as red numbers (**a**), more than 1 kcal/mol and less than 3 kcal/mol are shown as blue numbers (**b**), and less than 1 kcal/mol are shown as black numbers (**c**). Based on the position vector of Cα in Pin1 (PDB ID: 1PIN), the threshold of residue-residue distance was set to 15 Å. The domains presented here are WW domain (WW), catalytic loop (Cat. loop), α1-helix (α1), α2-3 helices (α2-3), and PPIase core.

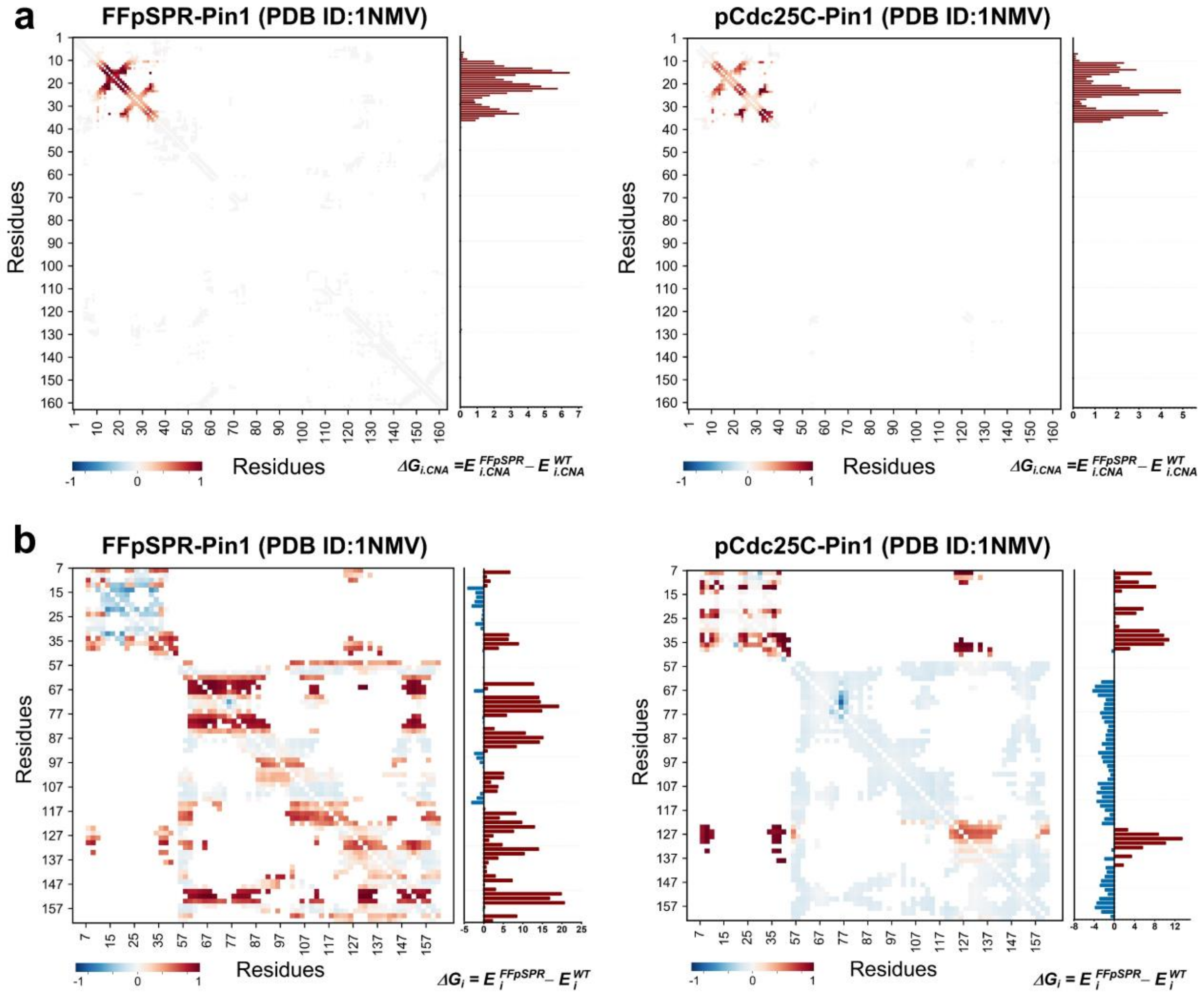

Supplementary Figure 35: Comparison of residue-residue interaction maps obtained by constraint network analysis (CNA) (**a**) and the GNN-based NRI model (**b**). The interaction maps reflect the changes in structural stability and edge weights due to the FFpSPR and pCdc25C binding. The histograms in (**a**) show the per-residue energy $\Delta G_{i,CNA}$ (Eq. (4) in Supplementary Note 3). The histograms in (**b**) show the per-residue weight $\Delta G_{i,Z}$ (Eq. (13) in the Supplementary Note 6). Based on the position vector of Cα in Pin1 (PDB ID:1NMV), the threshold of residue-residue distance is set to 12 Å to remove the interactions between two structurally non-neighboring residues.

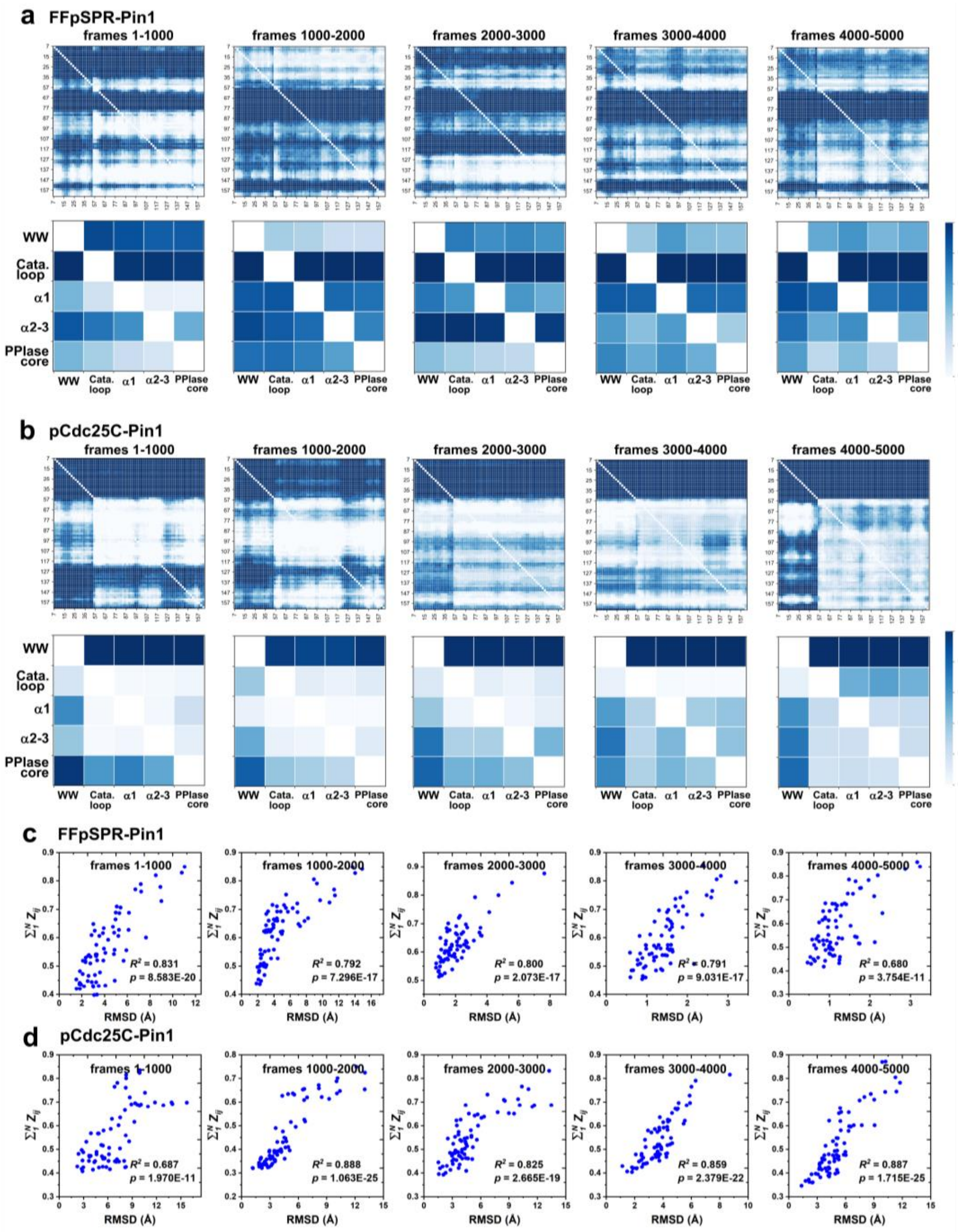

Supplementary Figure 36: The distributions of learned edges between residues and corresponding domains for the FFpSPR-bound (**a**) and pCdc25C-bound Pin1 (**b**). Correlation analysis between average per-residue RMSD value calculated from trajectories and per-residue weight value $\sum_1^N Z_{ij}$ learned by the NRI model (N represents the number of total residues) for the FFpSPR-bound (**c**) and pCdc25C-bound Pin1 (**d**). The edges are learned from frames 1-1000, 1000-2000, …, 4000-5000 of trajectories. The Pearson coefficient of correlation R^2 and p values are depicted in the legend. The p value was computed by two-sided test. The domains presented here are WW domain (WW), catalytic loop (Cata. loop), α1-helix (α1), α2-3 helices (α2-3), and PPIase core.

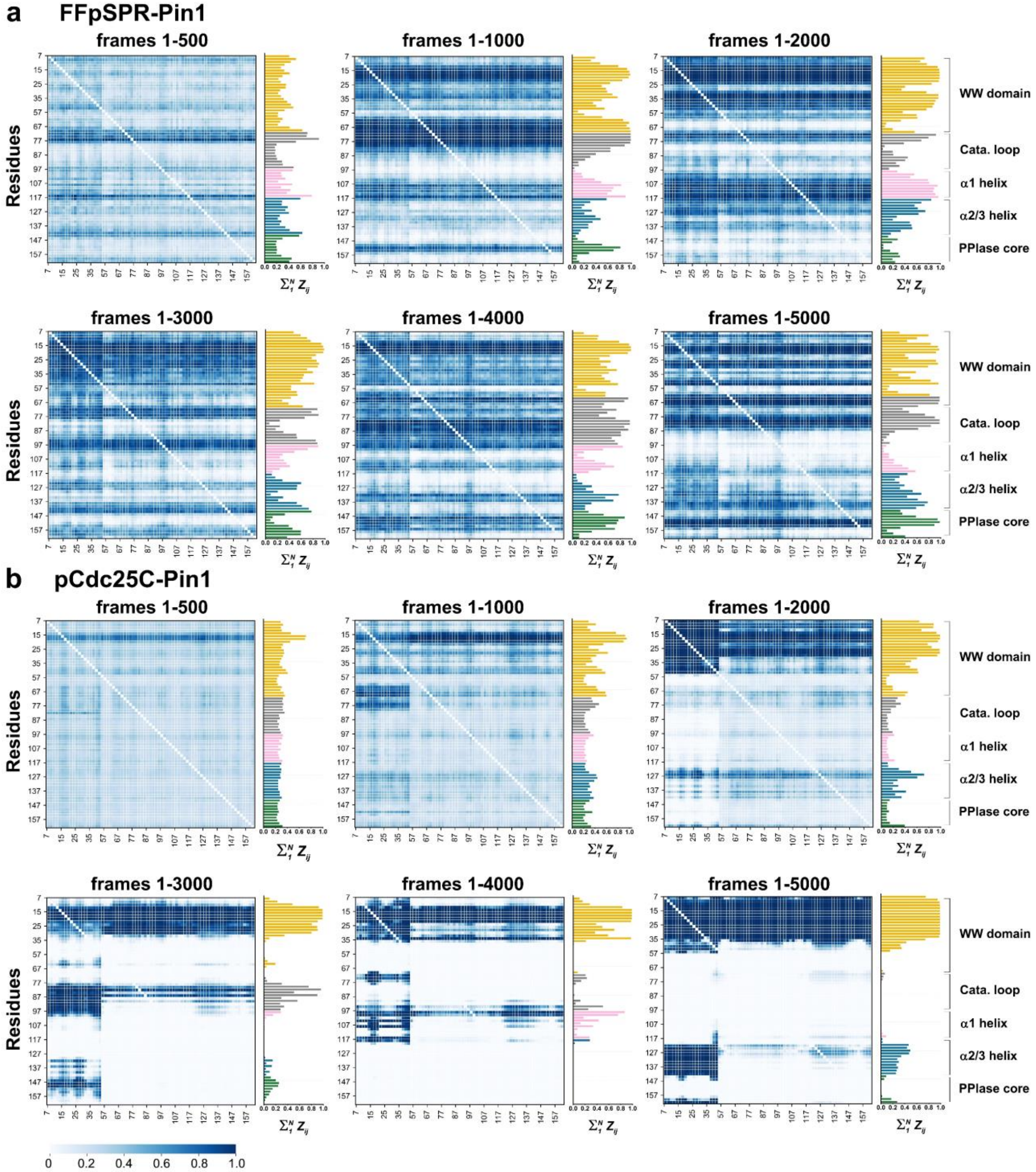

Supplementary Figure 37: The distributions of learned edges between residues for the FFpSPR-bound (**a**) and pCdc25C-bound Pin1 (**b**). The edges are learned from frames 1-500, 1-1000, …, 1-5000 of trajectories. The histograms show the per-residue weights $\sum_1^N Z_{ij}$ learned by the NRI model (N represents the number of total residues).

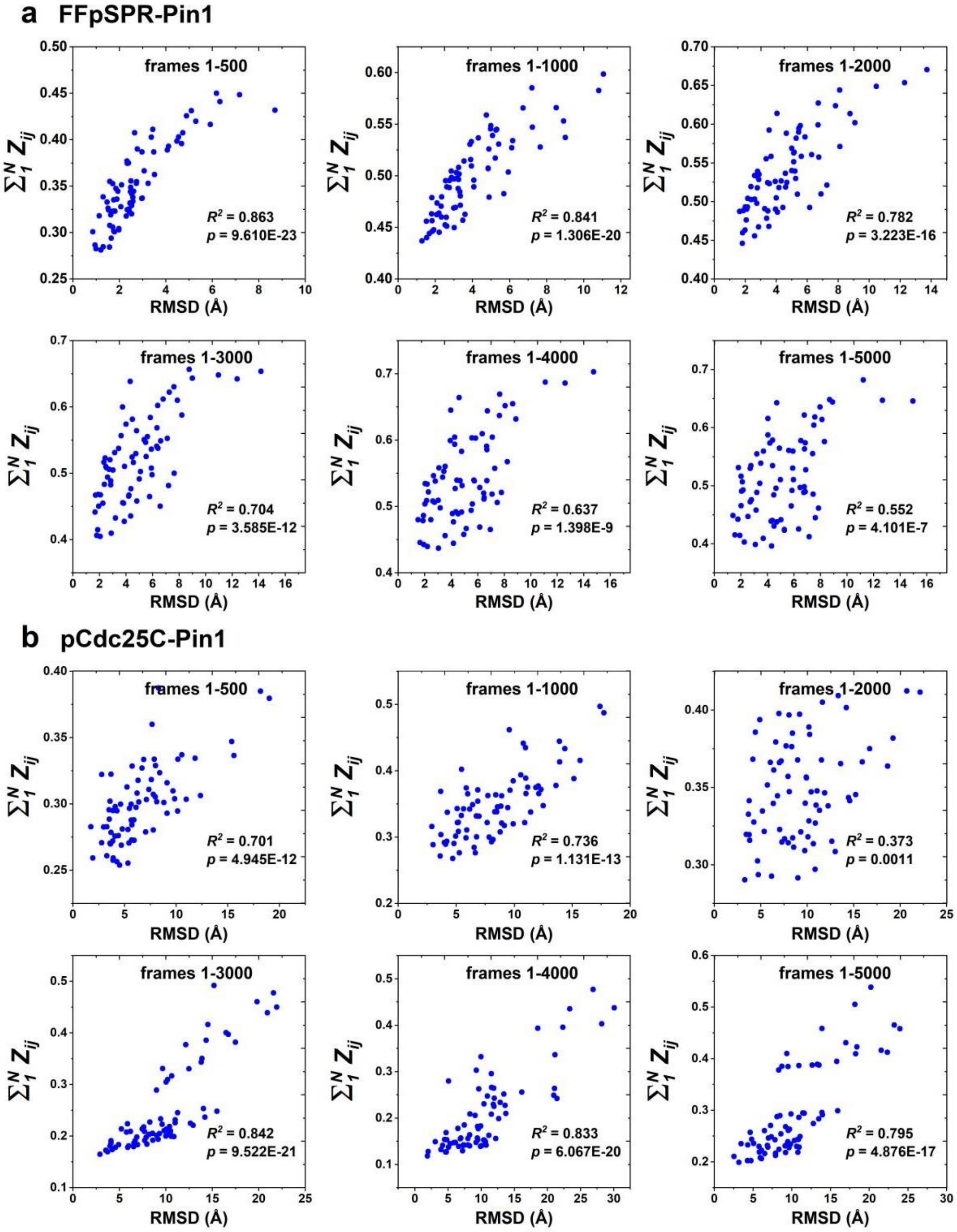

Supplementary Figure 38: Correlation analysis between average per-residue RMSD value calculated from trajectories and per-residue weight value $\sum_1^N Z_{ij}$ learned by the NRI model (N represents the number of total residues) for the FFpSPR-bound (**a**) and pCdc25C- bound Pin1 (**b**). The edges are learned from frames 1-500, 1-1000, …, 1-5000 of trajectories. The Pearson coefficient of correlation R^2 and p values are depicted in the legend. The p value was computed by two-sided test.

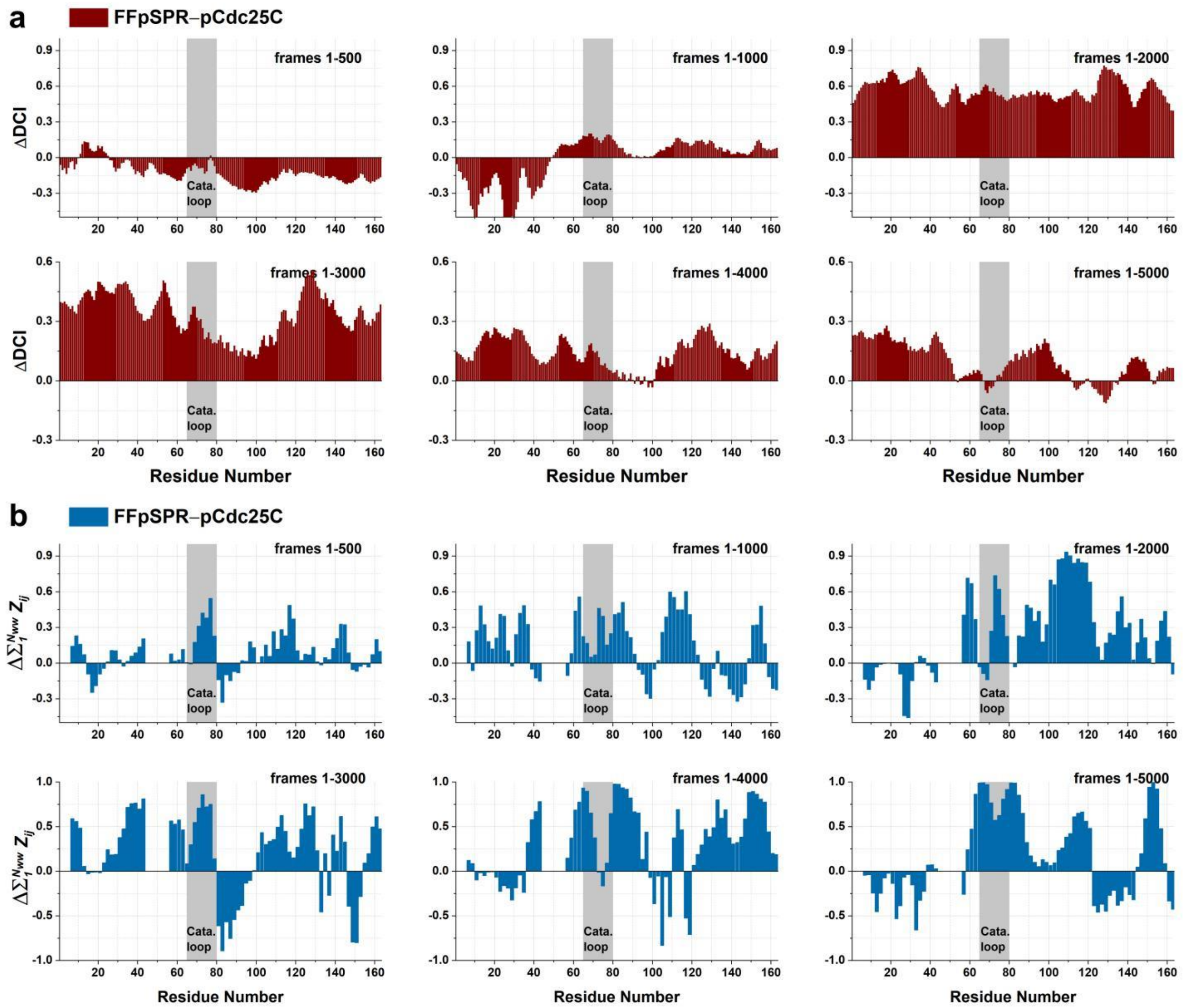

Supplementary Figure 39: Comparison of dynamics coupling index (DCI) analysis (**a**) and the NRI-based approach (**b**). The histograms in (**a**) show the difference of DCI value between FFpSPR-bound Pin1 and pCdc25C-bound Pin1. The histograms in (**b**) show the difference of per-residue weights $\sum_1^{N_{WW}} Z_{ij}$ between FFpSPR-bound Pin1 and pCdc25C-bound Pin1(N represents the number of total residues).

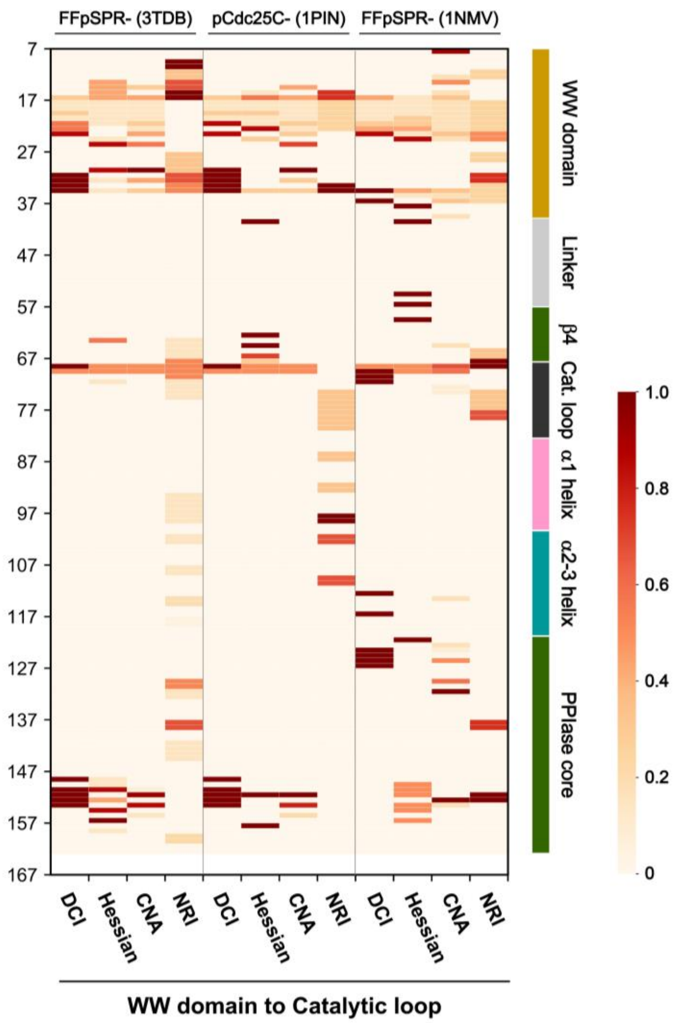

Supplementary Figure 40: The node centralities in the allosteric pathways between the WW domain and the catalytic loop for FFpSPR-bound Pin1 (closed conformation), pCdc25C-bound Pin1 (closed conformation), and FFpSPR-bound Pin1 (open conformation). The node centrality is calculated based on the covariance matrices obtained from DCI method, Hessian matrix, CNA method, and the NRI model, respectively. The color scale bar (from 1.0->0.0) represents the decrease in the importance of a residue measured by normalized node centrality (i.e., the fraction of suboptimal paths going through the node or residue). The main domains of Pin1 are consist of the WW domain (WW), β4-sheet (β4), catalytic loop (Cat. loop), α1-helix (α1), α2-3 helices (α2-3), and PPIase core.

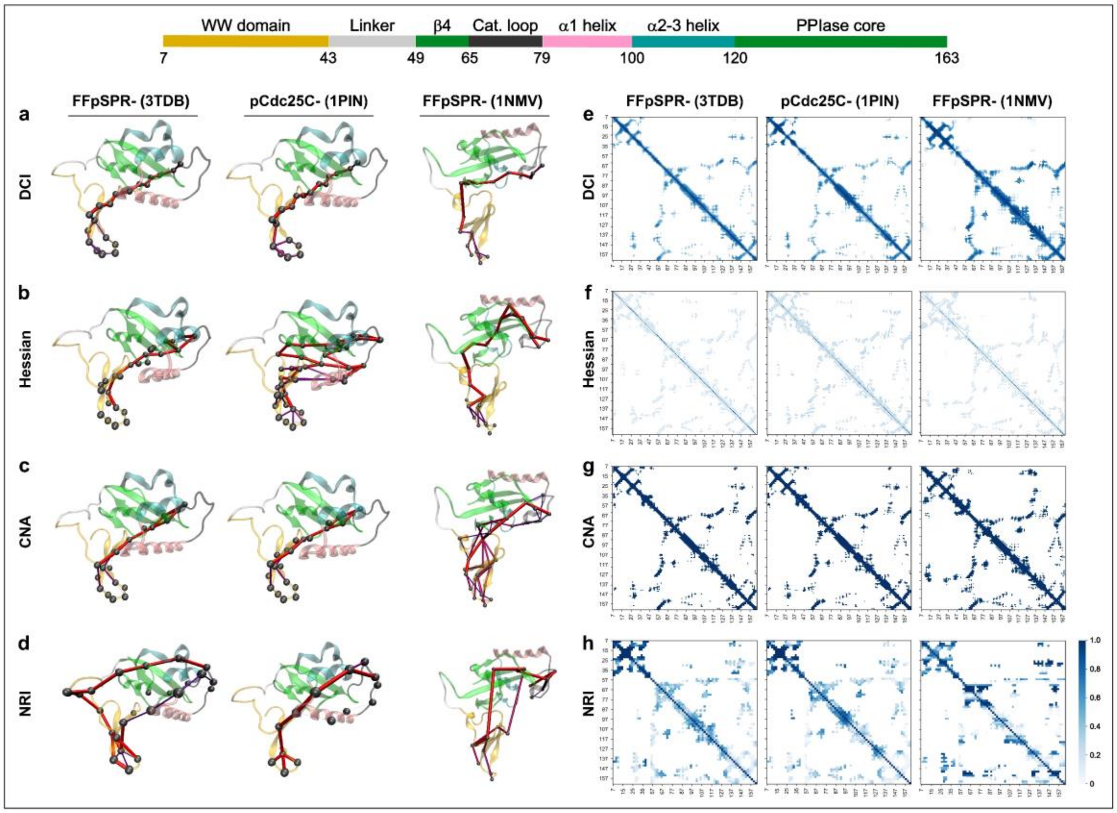

Supplementary Figure 41: The allosteric pathways mapped on the Pin1 structures for FFpSPR-bound Pin1 (closed conformation), pCdc25C-bound Pin1 (closed conformation), and FFpSPR-bound Pin1 (open conformation) obtained by DCI method (**a**), Hessian matrix (**b**), CNA method (**c**), and the NRI model (**d**). The covariance matrices obtained from DCI method (**e**), Hessian matrix (**f**), CNA method (**g**), and the NRI model (**h**). The main domains of Pin1 are consist of the WW domain (WW), β4-sheet (β4), catalytic loop (Cat. loop), α1-helix (α1), α2-3 helices (α2-3), and PPIase core.

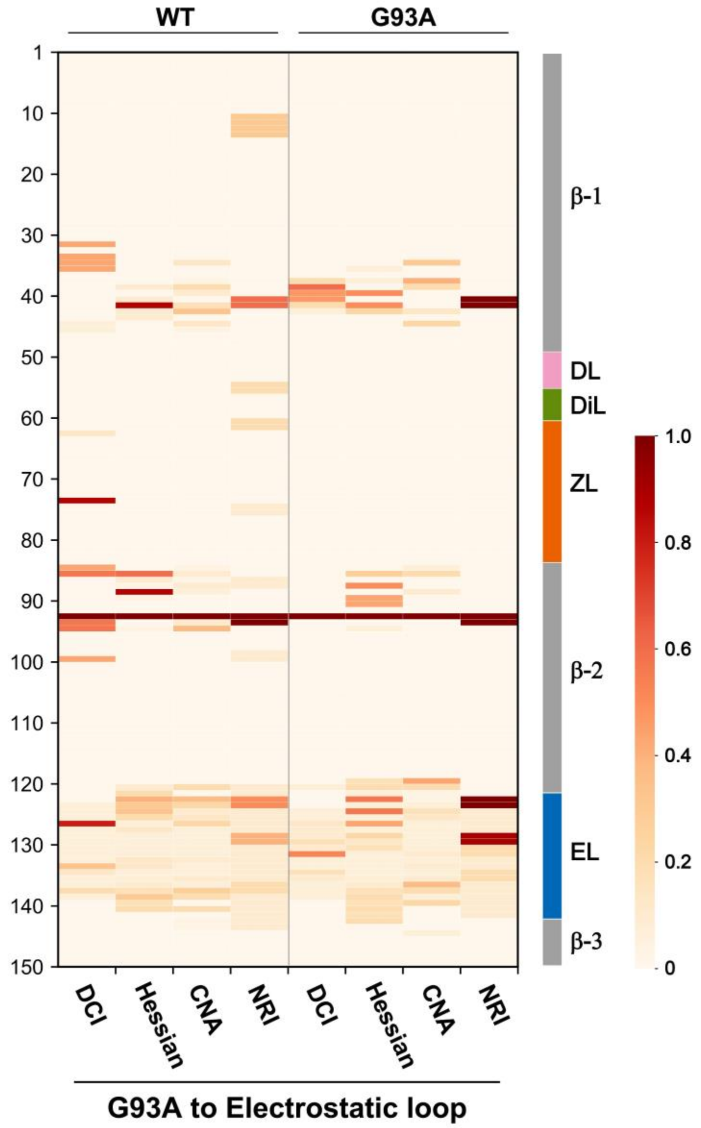

Supplementary Figure 42: The node centralities in the allosteric pathways between residue G93/A93 and the electrostatic loop for WT- and G93A-SOD1. The node centrality is calculated based on the covariance matrices obtained from DCI method, Hessian matrix, CNA method, and the NRI model. The main domains of SOD1 are consist of the β-barrel (β1-3), Dimerization loop (DL), Disulfide loop (DiL), Zinc loop (ZL), and Electrostatic loop (EL).

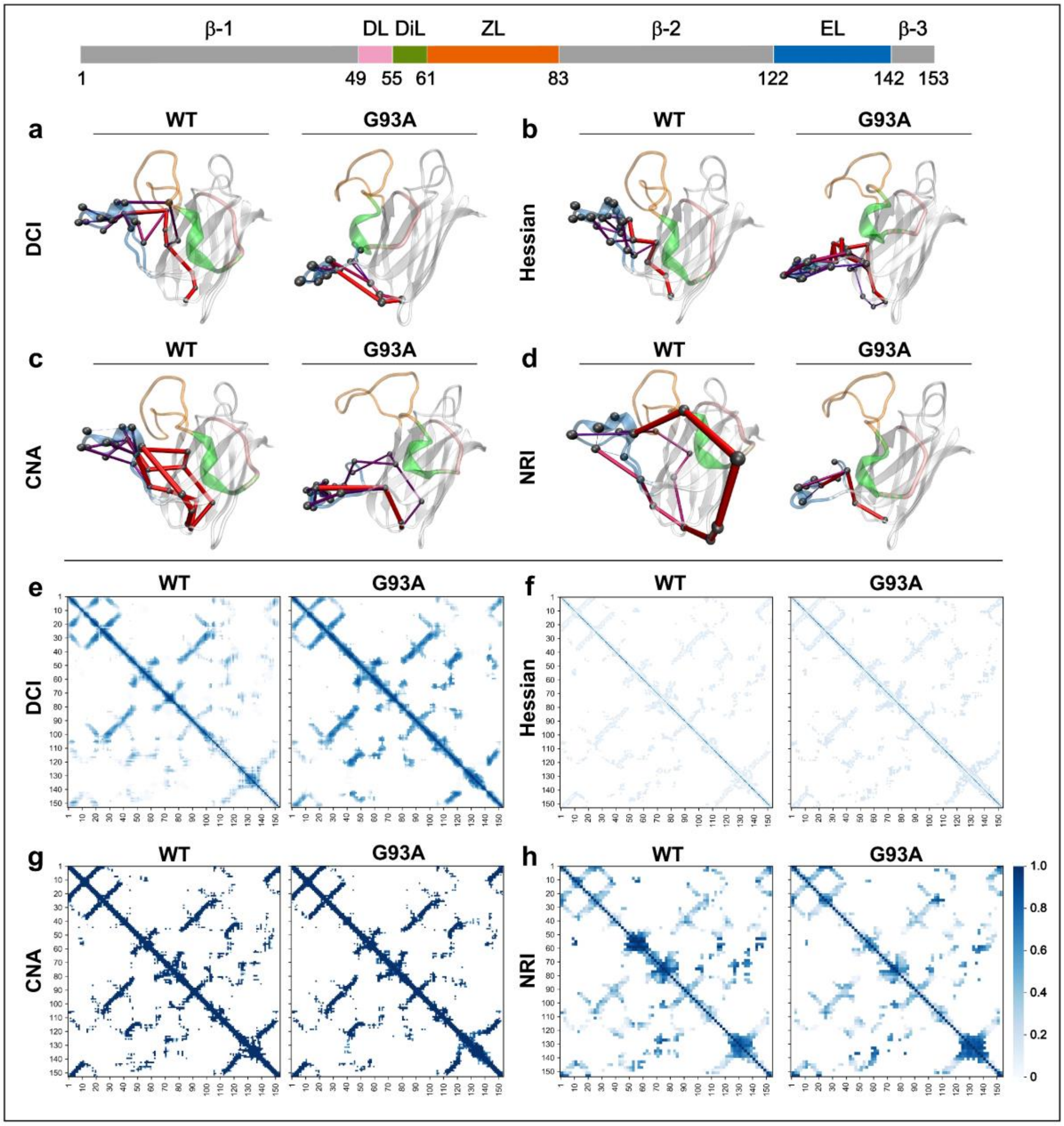

Supplementary Figure 43: The allosteric pathways mapped on the SOD1 structures for WT- and G93A-SOD1 obtained by DCI method (**a**), Hessian matrix (**b**), CNA method (**c**), and the NRI model (**d**). The covariance matrices obtained from DCI method (**e**), Hessian matrix (**f**), CNA method (**g**), and the NRI model (**h**). The main domains of SOD1 are consist of the β-barrel (β1-3), Dimerization loop (DL), Disulfide loop (DiL), Zinc loop (ZL), and Electrostatic loop (EL).

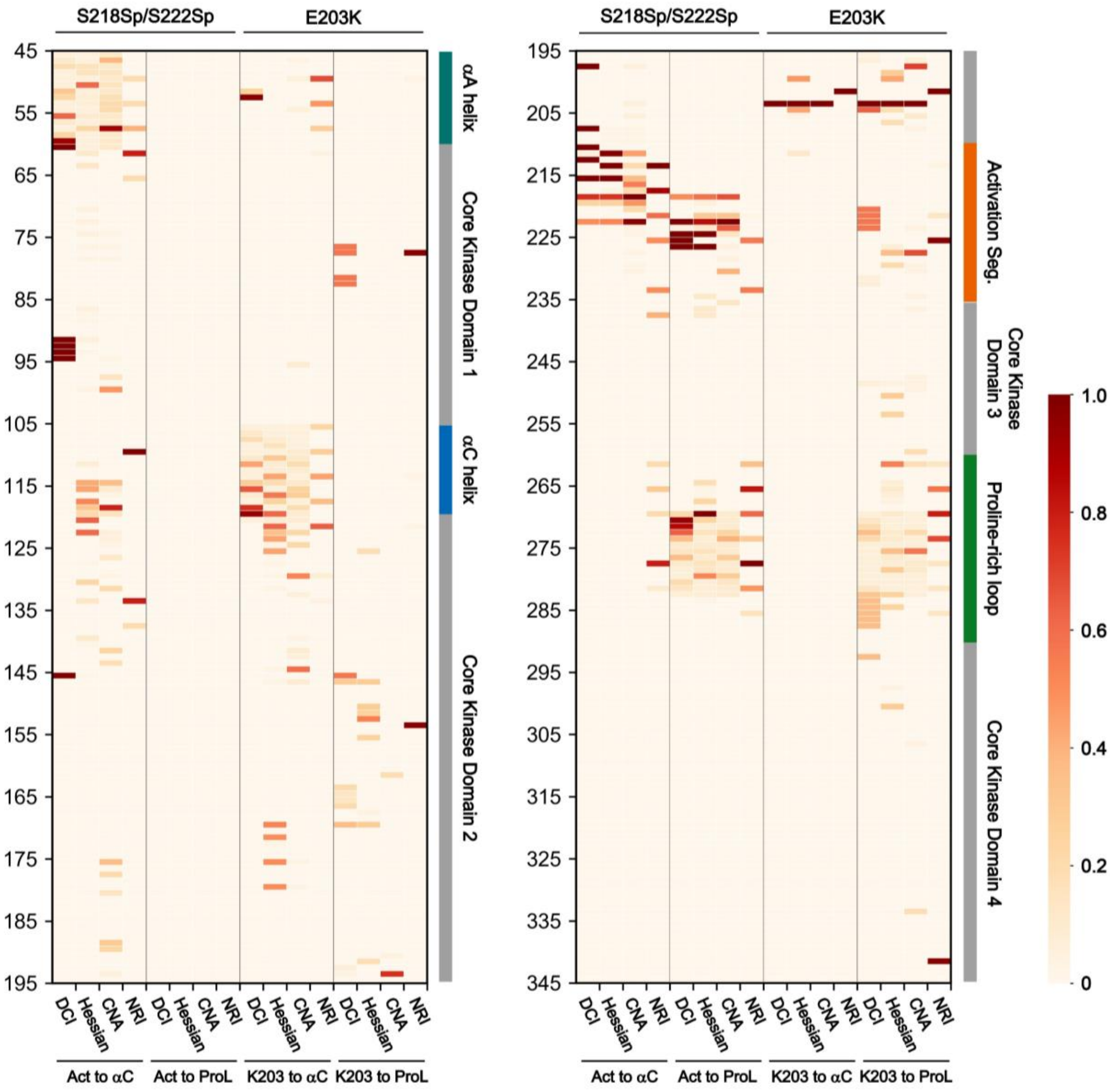

Supplementary Figure 44: The node centralities in the allosteric pathways between the activation segment and the αC-helix/proline-rich loop for S218Sp/S222Sp- and E203K-MEK1 complexes. The node centrality is calculated based on the covariance matrices obtained from DCI method, Hessian matrix, CNA method, and the NRI model.

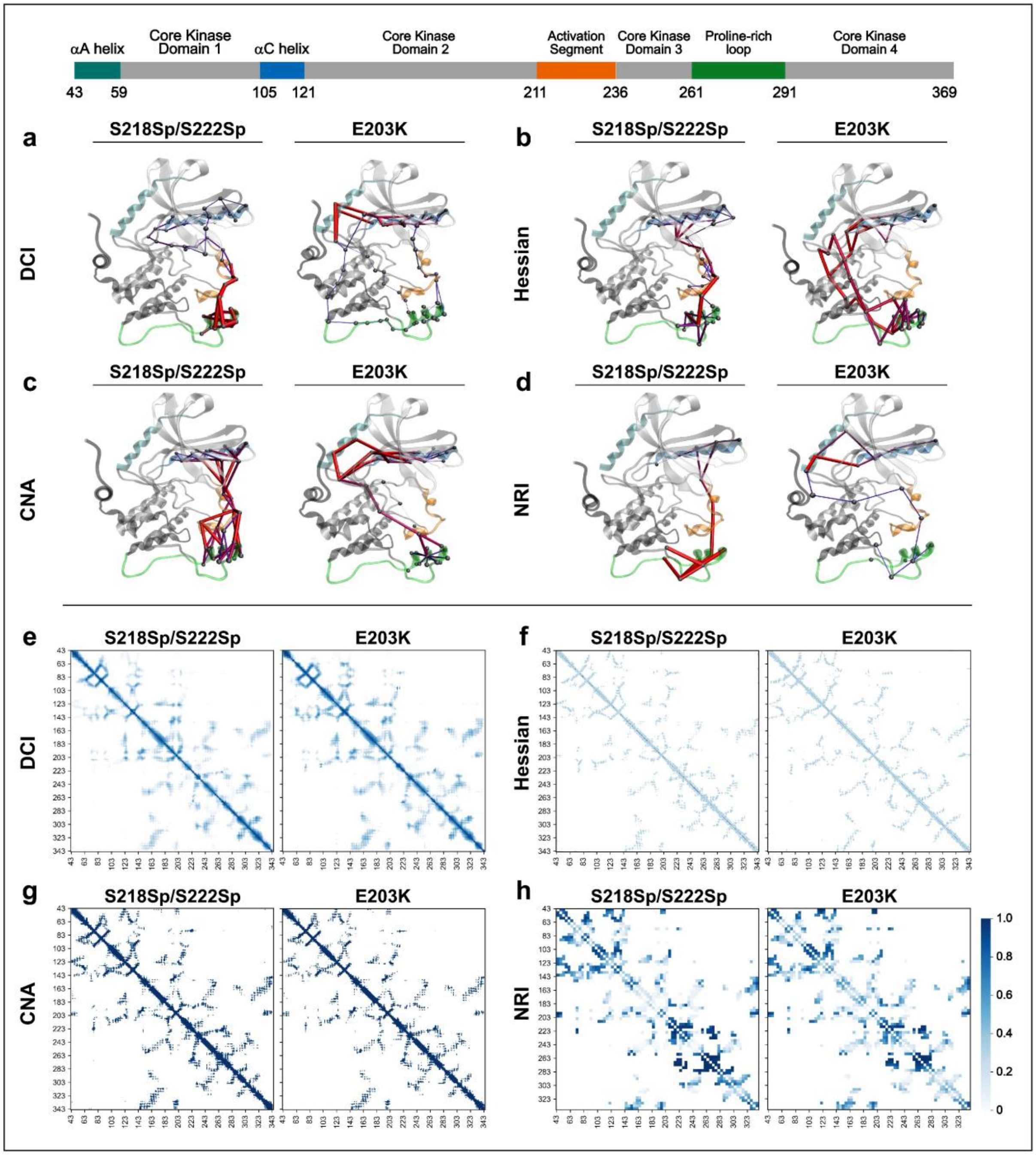

Supplementary Figure 45: The allosteric pathways mapped on the MEK1 structures for S218Sp/S222Sp- and E203K-MEK1 complexes, obtained by DCI method (**a**), Hessian matrix (**b**), CNA method (**c**), and the NRI model (**d**). The covariance matrices obtained from DCI method (**e**), Hessian matrix (**f**), CNA method (**g**), and the NRI model (**h**).

Supplementary Table 1: Shortest pathways from the residues in the WW domain to residues in the catalytic loop for the FFpSPR-Pin1, from the residues in the WW domain to K97 in the α1-helix for Apo-Pin1 and the FFpSPR-Pin1 (I28A), and from the residue in the WW domain to residues in the PPIase core for pCdc25C-Pin1.

FFpSPR-Pin1 (PDB ID: 3TDB)
L7>Y23>M15>E35>K97>S105>C113>K77
W11>M15>E35>K97>S105>C113>K77
K13>R21>M15>E35>K97>S105>C113>K77
M15>E35>K97>S105>C113>K77
R17>R21>M15>E35>K97>S105>C113>K77
S19>R21>M15>E35>K97>S105>C113>K77
R21>M15>E35>K97>S105>C113>K77
Y23>M15>E35>K97>S105>C113>K77
F25>R21>M15>E35>K97>S105>C113>K77
Q33>M15>E35>K97>S105>C113>K77
E35>K97>S105>C113>K77
P37>M15>E35>K97>S105>C113>K77
S41>P37>M15>E35>K97>S105>C113>K77
L7>Y23>M15>R17>R21>K13>T29>Q131>R69
P9>S41>P37>M15>R17>R21>K13>T29>Q131>R69
W11>M15>R17>R21>K13>T29>Q131>R69
K13>T29>Q131>R69
M15>R17>R21>K13>T29>Q131>R69
R17>R21>K13>T29>Q131>R69
S19>K13>T29>Q131>R69
R21>K13>T29>Q131>R69
Y23>M15>R17>R21>K13>T29>Q131>R69
F25>R21>K13>T29>Q131>R69
H27>M15>R17>R21>K13>T29>Q131>R69
T29>Q131>R69
A31>M15>R17>R21>K13>T29>Q131>R69
Q33>M15>R17>R21>K13>T29>Q131>R69
E35>M15>R17>R21>K13>T29>Q131>R69
P37>M15>R17>R21>K13>T29>Q131>R69
G39>S43>S41>P37>M15>R17>R21>K13>T29>Q131>R69
S41>P37>M15>R17>R21>K13>T29>Q131>R69
S43>S41>P37>M15>R17>R21>K13>T29>Q131>R69
L7>Y23>M15>R17>R21>K13>P9>P133>S67
P9>P133>S67
W11>M15>R17>R21>K13>P9>P133>S67
K13>P9>P133>S67
M15>R17>R21>K13>P9>P133>S67
R17>R21>K13>P9>P133>S67
S19>K13>P9>P133>S67
R21>K13>P9>P133>S67
Y23>M15>R17>R21>K13>P9>P133>S67
F25>R21>K13>P9>P133>S67
H27>M15>R17>R21>K13>P9>P133>S67
T29>Q131>S67
A31>M15>R17>R21>K13>P9>P133>S67
Q33>M15>R17>R21>K13>P9>P133>S67
E35>M15>R17>R21>K13>P9>P133>S67
P37>M15>R17>R21>K13>P9>P133>S67
G39>S43>S41>P37>M15>R17>R21>K13>P9>P133>S67
S41>P37>M15>R17>R21>K13>P9>P133>S67
S43>S41>P37>M15>R17>R21>K13>P9>P133>S67

Apo-Pin1 (PDB ID: 3TDB)
L7>E35>K97
P9>L7>E35>K97
W11>L7>E35>K97
K13>R17>Y23>S19>R21>E35>K97
M15>R21>E35>K97
R17>Y23>S19>R21>E35>K97
S19>R21>E35>K97
R21>E35>K97
Y23>S19>R21>E35>K97
F25>R21>E35>K97
H27>L7>E35>K97
T29>L7>E35>K97
A31>L7>E35>K97
Q33>M15>R21>E35>K97
P37>M15>R21>E35>K97
G39>L7>E35>K97
S41>G39>L7>E35>K97
S43>G39>L7>E35>K97

FFpSPR-Pin1 (PDB ID: 1NMV)
L7>T29>Q131>R69>D153>T81>K77
P9>T29>Q131>R69>D153>T81>K77
W11>T29>Q131>R69>D153>T81>K77
K13>T29>Q131>R69>D153>T81>K77
M15>H27>Q131>R69>D153>T81>K77
R17>M15>H27>Q131>R69>D153>T81>K77
S19>M15>H27>Q131>R69>D153>T81>K77
R21>K13>T29>Q131>R69>D153>T81>K77
Y23>H27>Q131>R69>D153>T81>K77
F25>T29>Q131>R69>D153>T81>K77
H27>Q131>R69>D153>T81>K77
T29>Q131>R69>D153>T81>K77
A31>T29>Q131>R69>D153>T81>K77
E35>S41>P9>T29>Q131>R69>D153>T81>K77
P37>T29>Q131>R69>D153>T81>K77
G39>T29>Q131>R69>D153>T81>K77
S41>P9>T29>Q131>R69>D153>T81>K77
S43>S41>P9>T29>Q131>R69>D153>T81>K77
L7>S43>S41>P9>S71
W11>S41>P9>S71
K13>S41>P9>S71
M15>H27>P9>S71
R17>K13>S41>P9>S71
S19>M15>H27>P9>S71
R21>K13>S41>P9>S71
Y23>S41>P9>S71
F25>S41>P9>S71
H27>P9>S71
T29>Q131>R69>D153>S65>S67>S71
A31>S41>P9>S71
E35>S41>P9>S71
P37>S43>S41>P9>S71
G39>T29>Q131>R69>D153>S65>S67>S71
S41>P9>S71
S43>S41>P9>S71

FFpSPR-Pin1(I28A) (PDB ID: 3TDB)
L7>K13>M15>R21>E35>K97
P9>K13>M15>R21>E35>K97
W11>M15>R21>E35>K97
K13>M15>R21>E35>K97
M15>R21>E35>K97
R17>M15>R21>E35>K97
S19>R21>E35>K97
R21>E35>K97
Y23>R21>E35>K97
F25>M15>R21>E35>K97
H27>M15>R21>E35>K97
T29>K13>M15>R21>E35>K97
A31>M15>R21>E35>K97
Q33>M15>R21>E35>K97
E35>K97
P37>M15>R21>E35>K97
G39>F25>M15>R21>E35>K97
S41>P37>M15>R21>E35>K97
S43>G39>F25>M15>R21>E35>K97

pCdc25C-Pin1(PDB ID: 1NMV)
L7>F125>P9>R127>P133
P9>R127>P133
W11>R127>P133
K13>H27>P9>R127>P133
M15>W11>R127>P133
R17>M15>W11>R127>P133
S19>M15>W11>R127>P133
R21>R17>M15>W11>R127>P133
Y23>W11>R127>P133
F25>H27>P9>R127>P133
H27>P9>R127>P133
T29>H27>P9>R127>P133
A31>H27>P9>R127>P133
Q33>H27>P9>R127>P133
E35>A31>H27>P9>R127>P133
P37>R127>P133
G39>S43>E35>A31>H27>P9>R127>P133
S41>E35>A31>H27>P9>R127>P133
S43>E35>A31>H27>P9>R127>P133

Supplementary Table 2: Probability of hydrogen-bond formation in the trajectories of WT and G93A of SOD1.

Donor	Acceptor	WT (%)	G93A (%)
L38: N	G93/A93: O	11.52	5.09
H43: NE2	T39: O	23.54	28.05
G44: N	H120: O	20.57	17.35
K122: NZ	A140: O	6.82	1.38
R115: NH1	E49: O	23.57	5.79
Q22: NE2	S105: OG	30.06	15.58
V47: N	G82: O	36.67	24.64
T116: OG1	F50: O	67.29	45.84
D124: N	N86: OD1	26.18	17.85
R79: NH2	D101: OD1	24.65	20.17

Supplementary Table 3: Shortest pathways from G93/A93 to the residues in the EL for WT and G93A of SOD1.

WT	G93A
G93>G41>D125	A93>A123>E133>D125
G93>A123>N131>G127	A93>A123>E133>G127
G93>A123>G129	A93>A123>E133>G129
G93>A123>N131	A93>A123>N131
G93>A123>N131>E133	A93>A123>E133
G93>V87>K75>T135	A93>A123>E133>T135
G93>K9>A55>C57>T137	A93>A123>T137
G93>A123>N131>N139	A93>V87>N139
G93>P13>C57>A55>S59>G141	A93>V87>G141

Supplementary Table 4: Shortest pathways from N221 in the activation segment to the residues in the αA-helix and proline-rich loop for S218Sp/S222Sp MEK1, and from R201 (near E203K) to the residues in the αC-helix and proline-rich loop for E203K MEK1.

S218Sp/S222Sp	E203K
N221>D217>G213>R113>C121>R49	R201>Q45>R49>K57>V117>N109>P105
N221>D217>G213>N109>F133>G61>F53	R201>Q45>R49>K57>V117>N109
N221>D217>G213>V117>K57	R201>Q45>R49>K57>V117>R113
N221>D217>G213>N109>F133>G61	R201>Q45>R49>K57>V117
N221>D217>G213>N109>G137>D65	R201>Q45>R49>C121
N221>D277>S265	R201>Q45>R49>K57>V117>G213>D217>G237>F273>S265>Y261
N221>D277>S265>S269	R201>Q45>R49>K57>V117>G213>D217>G237>F273>S265
N221>D277>S265>F273	R201>Q45>R49>K57>V117>G213>D217>G237>F273>S269
N221>D277>N281	R201>Q45>R49>K57>V117>G213>D217>G237>F273
N221>D277>S265>S269>P285	R201>Q45>R49>K57>V117>G213>D217>G237>D277
N221>D277>S265>S269>P285>S289	R201>Q45>R49>K57>V117>G213>D217>G237>N281
N221>D277>S265>S269>P285>S289>S293	R201>F333>E329>S293
N221>D277>S265>S269>P285>Q297	

Supplementary Table 5: Mean squared error (MSE) values for the proposed model and the model without latent variables on edges.

	**Apo-Pin1-3TDB**	**WT-MEK1**	**WT-SOD1**
Proposed	0.00478	0.00176	0.00430
No latent variables on edges	0.00498	0.00189	0.01111

Supplementary Note 1. Simulation data

1) Preparation of protein structures

The crystal structures for the three systems (Pin1, SOD1, and MEK1) were retrieved from the Protein Data Bank (https://www.rcsb.org/). The apo Pin1 structure was obtained from PDB 3TDB (http://doi.org/10.2210/pdb3TDB/pdb). To obtain the Pin1-FFpSPR complex, we docked the substrate (FFpSPR) into the WW-domain of apo Pin1 using Autodock 4.2[1]. Missing residues of the protein (residues 39-50) in the inter-domain linker were modeled by SWISS-MODEL[2]. In the negative regulation study, the closed pCdc25C-Pin1 structure was obtained from PDB 1PIN (http://doi.org/10.2210/pdb1PIN/pdb), the open Pin1 structure with the WW and PPIase domains well-separated was obtained from the first model of PDB 1NWV (http://doi.org/10.2210/pdb1NMV/pdb). To obtain the FFpSPR- and pCdc25C-bound complexes, we docked these two substrates into the WW-domain using Autodock 4.2. In the free energy score calculation study, the Pin1 structure was obtained from PDB 1PIN, and the corresponding 23 Ala-mutants were modeled by SWISS-MODEL server. The SOD1 and MEK1 structures were taken directly from PDB 2C9V (http://doi.org/10.2210/pdb2C9V/pdb) and 3SLS (http://doi.org/10.2210/pdb3SLS/pdb). The structures of corresponding mutants were also constructed using the SWISS-MODEL server.

2) Conventional molecular dynamics (cMD) simulations

The cMD simulations of four MEK1 structures (WT, A52V, E203K, and phosphorylated MEK1) were performed using the GROMACS 5.1.4 package with the 53A6 GROMOS force field[3]. All complexes of MEK1 structures and an A-type natriuretic peptide (ANP), which was used as an inhibitor, were performed in a periodic boundary box with the simple point charge (SPC) water model[4]. The distance between the solute surface and the box was set to 10 Å. Two sodium ions were added to the box to neutralize the WT and A52V MEK1 systems, and four sodium ions were added to the box to neutralize the S218Sp/S222Sp MEK1 system. Note that the E203K MEK1 system does not need to add ions for neutralization. In addition, energy minimization was performed using the steepest descent method to obtain the energy-minimized initial structure for the next simulations. Subsequently, 100 ps of NVT (Berendsen temperature coupled with constant particle number, volume, and temperature)[5] and 100 ps of NPT (Parrinello-Rahman pressure coupled with constant particle number, pressure, and temperature)[5] were performed to maintain the stability of the system (300 K, 1 bar). The coupling constants for temperature and pressure were set at 0.1 and 2.0 ps, respectively. Long-range electrostatic interactions were described using the particle mesh Ewald (PME) algorithm[6] with an interpolation order of 4 and a grid spacing of 1.6 Å, van der Waals interactions were calculated according to the cutoff value of 12 Å. All bond lengths were constrained using the LINear Constraint Solver (LINCS) algorithm[6]. After stabilizing all thermodynamic properties, the molecular systems were simulated for 200 ns with a time interval of 2 fs.

3) Gaussian accelerated molecular dynamics (GaMD) simulation

GaMD simulation is an enhanced sampling technique performed by adding a harmonic boost potential to smoothen the system's potential energy surface[7, 8]. To enhance the conformational sampling, GaMD simulations were performed on the Pin1, SOD1 and MEK1 structures. For each

system, the graphic processing unit (GPU) version of AMBER18 was applied to perform GaMD simulation[9]. All simulations were performed in a periodic boundary box with TIP3P water model[4]. The distance between the solute surface and the box was set to 10 Å. The Amber ff14SB force field[10] was used to generate force field parameters of protein and peptide. The generalized AMBER force field (GAFF) parameters[11] and RESP partial charges[11] were assigned for the ligands in complexes. The SHAKE algorithm was used to constrain all bonds involving hydrogen atoms[12]. The particle mesh Ewald (PME) algorithm[6] was used to handle non-bonded electrostatic interactions with the 10 Å cutoff distance. Before the GaMD simulation, energy minimization with the steepest descent algorithm and conjugate gradient algorithm was executed to eliminate atomic collisions in the initial structure. The systems were gradually heated to 300 K under NVT ensemble, and were equilibrated under the NPT ensemble. The GaMD simulation has five stages: (i) conventional MD preparatory stage for the equilibration of the system, (ii) conventional MD stage to collect potential statistics for calculating the GaMD acceleration parameters, (iii) GaMD pre-equilibration stage with boost potential, (iv) GaMD equilibration stage to update the boost parameters, and (v) multiple independent GaMD production runs with randomized initial atomic velocities. We repeated the simulations for these three systems twice to evaluate the robustness of the NRI model. For the closed Pin1 structures (Apo-Pin1, FFpSPR-Pin1, and FFpSPR-Pin1(I28A) obtained from PDB 3TDB), the first simulation was run 200 ns, and the last two simulations were run 500 ns. For the open Pin1 structures (FFpSPR-Pin1 and pCdc25C-Pin1 obtained from PDB 1NMV), all three simulations were run 500 ns. For the closed pCdc25C-Pin1 structure (obtained from PDB 1PIN), the simulation was run 500 ns. For the wild type (WT) and 23 Ala-mutants of unbound Pin1 (obtained from PDB 1PIN), all the simulations were run 500 ns. For the SOD1 system, the first simulation was run 300 ns, the last two simulations were run 500 ns. For the MEK1 system, the first simulation was run 200 ns, the last two simulations were run 1 us. The GaMD simulation trajectories were analyzed using CPPTRAJ from AmberTools16[13] and VMD[14] for RMSF calculation, secondary structure analysis, principal component analysis (PCA), hydrogen bond calculation, and Dynamical Cross-Correlation Matrix (DCCM). To quantitatively evaluate the performance of our model, the trajectories were additionally analyzed by the constraint network analysis (CNA version 2.0)[15], derivative centrality metric of the Hessian[16], and dynamic coupling index (DCI) metric[17] (see Supplementary Note 3-5 for method details).

Supplementary Note 2. NRI model construction details

All NRI trainings were performed using Adam optimizer[18] with a learning rate of 0.0005 and a batch size of 1, decayed by a factor of 0.5 every 200 epochs. The concrete distribution was used with $\tau = 0.5$. During the testing, we replaced the concrete distribution with a categorical distribution to obtain discrete latent edge types. All experiments were run for 500 training epochs. The discrete samples were used in the training forward pass. We saved model checkpoints after every epoch whenever the validation set performance improved and loaded the best performing model for the test set evaluation. We used a standard Nvidia GeForce GTX 1080Ti GPU card and a Core solo CPU to train our models. Each CPU was allocated 48 GB memory. The training time for one experiment took about 5 hours.

We used the MD trajectories to generate the input data for the next training, validation, and test[19]. The dataset of the Pin1 system has a total size of 2000, 2500 or 5000 frames for 73 Cα atoms each. The dataset of the SOD1 system has 3000 or 5000 frames for 77 Cα atoms each.

The dataset of the MEK1 system has 1900, 2500 or 5000 frames for 74 Cα atoms each. We normalized the position and velocity features to the maximum absolute value of 1. The overall input/output dimension of the model is 6 (3D position and velocity). Training, validation, and test samples each contain 50 frames uniformly extracted from each trajectory. For the training part, the model received a ground truth input in each timestep. The dynamics for our three systems changed considerably over time, and the protein conformations in the early stage of the simulation are quite different from those in the end stage. Therefore, in the experimental tests, we fed in the 10, 15, 20, 25, 30, 40, 45, 50, 60, 75, 90, 100 frames as the ground truth to the encoder and then reconstructed these timesteps. All experiments used the multi-layer perceptron (MLP) encoder and recurrent neural network (RNN) decoder to have a capacity comparable to the full graph model. The first edge type is "hard-coded" as non-edge (no messages are passed along this type). The basic building block of the MLP encoder is a 2-layer MLP with a hidden and output (embedding) dimension of 256, together with batch normalization, dropout, and ELU activations. The RNN decoder adds a GRU-style[20] update to the single-step prediction. Given the interaction graph learned from the NRI model, we took the allosteric site as the starting point and the active site as the terminal point to calculate the shortest pathways using Dijkstra's algorithm[21].

Supplementary Note 3. Free energy quantity derived from mechanical stability (Constraint network analysis)

The neighbor stability maps ($rc_{ij,neighbor}$), which reflect the local stabilities of the residue-residue contacts, were obtained using the CNA software package[22]. The chemical potential energy due to noncovalent bonding can be calculated by summing over the contacts in the stability map, which takes the form

$$E_{CNA} = \sum_i^n \sum_{j>i}^n rc_{ij,neighbor} \quad (1)$$

The change in biomolecular stability upon ligand binding or a mutation reflects the difference in the chemical potential energy ΔE_{CNA}, i.e.,

$$\Delta E_{CNA} = E_{CNA,perturbed} - E_{CNA,ground} \quad (2)$$

which is detected in terms of an ensemble-based perturbation. In Equation (2), $E_{CNA,ground}$ and $E_{CNA,perturbed}$ represent the free energies of the ground and perturbed states. The ground state contains a conformational ensemble of network topologies with allosteric ligand binding or residue mutation. The perturbed state is obtained by removing the noncovalent and covalent interactions associated with ligand and residue mutation. The energy difference resulting from conformational changes due to a perturbation is excluded in this approach. The perturbation of a ground network is localized and small. Thus, a one-step free energy perturbation approach[23] is applied as an approximation to compute the free energy ΔG_{CNA},

$$\Delta G_{CNA} = -k_B T - ln \langle exp(-\frac{\Delta E_{CNA}}{k_B T})\rangle_{ground} \quad (3)$$

where k_B is the Boltzmann constant, and the temperature T is set to 300 K. $\langle \ldots \rangle_{ground}$ denotes averaging over the ground-state ensemble.

Per-residue free energy $\Delta G_{i,CNA}$ allows for identifying the residue's (node's) importance in residue interaction networks contributed to the allosteric signaling. Based on a linear response approximation[24], the per-residue decomposition is defined as:

$$\Delta G_{i,CNA} = \frac{1}{2}(\langle E_{i,CNA}^{perturbed}\rangle - \langle E_{i,CNA}^{ground}\rangle) \quad (4)$$

where $E_{i,CNA}$ represents the chemical potential energy of residue i obtained by summing over short-range rigid contacts. $E_{i,CNA}$ takes the form

$$E_{i,CNA} = \frac{1}{2}\sum_{j\neq i}^{n} rc_{ij,neighbor} \quad (5)$$

Supplementary Note 4. Derivative centrality metric of the Hessian

To calculate the derivative centrality metric[16], an effective harmonic Hessian needs to be obtained first using a slightly modified heterogeneous ENM (hENM) procedure[25, 26]. Specifically, an $N \times N$ force constant matrix, k, is constructed by the hENM procedure. The off-diagonal 3×3 tensor element of the $3N \times 3N$ Hessian matrix is defined as

$$H_{ij} = -k_{ij}\hat{R}_{ij} \otimes \hat{R}_{ij} \quad (6)$$

Then, the $3N \times 3N$ Hessian can be reconstructed as $C = TH^{+}$ by performing the Moore-Penrose pseudo inverse of H.

The application of derivative centrality metric is to identify the change of the covariance between a given set of sources and target nodes upon changing a single spring constant. An edge-based centrality metric is defined as

$$\delta_{edge}^{(ij)} = \frac{d\|C_{mn}\|^2}{dk_{ij}} \quad (7)$$

where $\|C_{mn}\|^2$ is the squared Frobenius norm of the covariance tensor between source m and target n. The node-based centrality metric is defined by mapping this edge-based metric, which takes the form

$$\delta_{node}^{(ij)} = \frac{\sum_i k_{ij}\delta_{edge}^{(ij)}}{max\left(\left\{\left|\sum_i k_{i'j'}\delta_{edge}^{(i'j')}\right|\right\}\right)} \quad (8)$$

which is motivated by considering a fractional change of all the spring constants connecting a given node.

Supplementary Note 5. Dynamic coupling index (DCI)

The DCI metric[17] allows for identifying residues that are distal to functional sites but remotely impact active site dynamics. Like the derivative centrality metric, the positional residue matric is obtained using ENM procedure[22, 26]. To model the changes in interaction networks more accurately upon ligand binding, the Hessian matrix is replaced with the covariance matrix obtained from MD simulations. The fluctuation response profile of the position due to the perturbation of a residue is obtained using the linear response theory[27] as follows:

$$[\Delta R_{3N\times 1}] = [G_{3N\times 3N}][F_{3N\times 1}] \quad (9)$$

DCI is defined as the ratio of the sum of the mean square fluctuation response of residue i upon functional site perturbations to the response of residue j upon perturbations on all residues,

$$\mathrm{DCI}_i = \frac{\sum_j^{N_{functional}} |\Delta R^j|_i / N_{functional}}{\sum_{j=1}^{N} |\Delta R^j|_i / N} \quad (10)$$

where $|\Delta R^j|_i$ is the fluctuation response profile of the residue i upon perturbation of residue j. The numerator is the average mean square fluctuation response obtained over the perturbation of the functionally critical residues $N_{functional}$, and the denominator is the average mean square fluctuation response over all residues.

Supplementary Note 6. Free energy score derived from the NRI modeling

The NRI-based free energy score is calculated using the factorized distribution of Z_{ij},

$$z_{i,j} = softmax((h^2_{(i,j)} + g)/\tau) \quad (11)$$

is learned from the NRI model training, representing the edge weight between node i and node j in the interacting network. To exclude edges of structurally non-neighboring residues, we focused on short-range contact by removing the edges between two nodes separated by more than 12 Å or 15 Å. Hence, the free energy score E_z,

$$E_z = \sum_i^n \sum_{j>i}^n Z_{ij,neighbor} \quad (12)$$

is calculated by summing over all edge weights. The difference in the free energy score $\Delta G_Z = E_z^{Ala-mutant} - E_z^{WT}$ reflects the change in residue interactions, because of the ligand binding or a residue mutation.

Per-residue free energy score $\Delta G_{i,Z}$ upon the ligand binding or a mutation is defined as:

$$\Delta G_{i,Z} = E_{i,Z}^{ligand/mutation} - E_{i,Z}^{Apo/WT} \quad (13)$$

where $E_{i,Z}$ represents the free energy score of the residue i obtained by summing over short-range interactions. It takes the form

$$E_{i,Z} = \sum_{j \neq i}^n Z_{ij,neighbor} \quad (14)$$

References

1. Morris, G. M. *et al.* AutoDock4 and AutoDockTools4: Automated docking with selective receptor flexibility. *J. Comput. Chem.* **30**, 2785-2791 (2009).
2. Biasini, M. *et al.* SWISS-MODEL: modelling protein tertiary and quaternary structure using evolutionary information. *Nucleic acids Res.* **42**, W252-258 (2014).
3. Oostenbrink, C., Soares, T. A., van der Vegt, N. F. & van Gunsteren, W. F. Validation of the 53A6 GROMOS force field. *Eur. Biophys. J.* **34**, 273-284 (2005).
4. Mark, P. & Nilsson, L. Structure and dynamics of the TIP3P, SPC, and SPC/E water models at 298 K. *J. Phys. Chem. A* **105**, 9954-9960 (2001).

5. Berendsen, H. J. C., Postma, J. P. M., van Gunsteren, W. F., DiNola, A. & Haak, J. R. Molecular dynamics with coupling to an external bath. *J. Chem. Phys.* **81**, 3684-3690 (1984).
6. Darden, T., York, D. & Pedersen, L. Particle mesh Ewald: An N·log(N) method for Ewald sums in large systems. *J. Chem. Phys.* **98**, 10089-10092 (1993).
7. Miao, Y., Feher, V. A. & McCammon, J. A. Gaussian accelerated molecular dynamics: Unconstrained enhanced sampling and free energy calculation. *J. Chem. Theory Comput.* **11**, 3584-3595 (2015).
8. Miao, Y. & McCammon, J. A. Gaussian accelerated molecular dynamics: Theory, implementation, and applications. *Annual reports in computational chemistry* **13**, 231-278 (2017).
9. Lee, T. S. *et al.* GPU-accelerated molecular dynamics and free energy methods in Amber18: Performance enhancements and new features. *J. Chem. Inf. Model.* **58**, 2043-2050 (2018).
10. Maier, J. A., Martinez, C., Kasavajhala, K., Wickstrom, L., Hauser, K. E. & Simmerling, C. ff14SB: Improving the Accuracy of Protein Side Chain and Backbone Parameters from ff99SB. *J. Chem. Theory Comput.* 11, 3696-3713 (2015).
11. Özpınar, G. A., Peukert, W., Clark, T. An improved generalized AMBER force field (GAFF) for urea. J. Mol. Model. 16, 1427-1440 (2010).
12. Ryckaert J. P., Ciccotti G., Berendsen H. J. C. Numerical integration of the cartesian equations of motion of a system with constraints: molecular dynamics of n-alkanes. J. Comput. Phys. 23, 327-341 (1977).
13. Roe, D. R. & Cheatham, T. E. PTRAJ and CPPTRAJ: Software for processing and analysis of molecular dynamics trajectory data. *J. Chem. Theory Comput.* **9**, 3084-3095 (2013).
14. Humphrey, W., Dalke, A. & Schulten, K. VMD: visual molecular dynamics. *J. Mol. Graph.* **14**, 33-38, 27-38 (1996).
15. Pfleger, C., Minges, A., Boehm, M., McClendon, C. L., Torella, R. & Gohlke, H. Ensemble- and rigidity theory-based perturbation approach to analyze dynamic allostery. *J. Chem. Theory Comput.* **13**, 6343-6357 (2017).
16. Lake, P. T., Davidson, R. B., Klem, H., Hocky, G. M. & McCullagh, M. Residue-level allostery propagates through the effective coarse-grained Hessian. *J. Chem. Theory Comput.* **16**, 3385-3395 (2020).
17. Campitelli, P., Guo, J., Zhou, H. X. & Ozkan, S. B. Hinge-shift mechanism modulates allosteric regulations in human Pin1. *J. Phys. Chem. B* **122**, 5623-5629 (2018).
18. Kingma, D. P. & Ba, J. L. Adam: A method for stochastic optimization. In: *International Conference on Learning Representations* (2015).
19. Zhu, J., Wang, J., Han, W. & Xu, D. Neural relational inference to learn long-range allosteric interactions in proteins from molecular dynamics simulations. *Zenodo* https://doi.org/10.5281/zenodo.5941385 (2022).
20. Cho, K. *et al.* Learning phrase representations using RNN encoder-decoder for statistical machine translation. In: *Empirical Methods in Natural Language Processing* (2014).
21. Dijkstra, E. W. A note on two problems in connexion with graphs. *Numer. Math.* **1**, 269-271 (1959).

22. Pfleger, C., Rathi, P. C., Klein, D. L., Radestock, S. & Gohlke, H. Constraint network analysis (CNA): A python software package for efficiently linking biomacromolecular structure, flexibility, (thermo-)stability, and function. *J. Chem. Inf. Model.* **53**, 1007-1015 (2013).
23. Zwanzig, R. W. High-temperature equation of state by a perturbation method. I. Nonpolar Gases. *J. Chem. Phys.* **22**, 1420-1426 (1954).
24. Lee, F. S., Chu, Z. T., Bolger, M. B. & Warshel, A. Calculations of antibody-antigen interactions: microscopic and semi-microscopic evaluation of the free energies of binding of phosphorylcholine analogs to McPC603. *Protein Eng. Des. Sel.* **5**, 215-228 (1992).
25. Gerek, Z. N. & Ozkan, S. B. Change in allosteric network affects binding affinities of PDZ domains: analysis through perturbation response scanning. *PLoS Comput. Biol.* **7**, e1002154 (2011).
26. Atilgan, A. R., Durell, S. R., Jernigan, R. L., Demirel, M. C., Keskin, O. & Bahar, I. Anisotropy of fluctuation dynamics of proteins with an elastic network model. *Biophys. J.* **80**, 505-515 (2001).
27. Ikeguchi, M., Ueno, J., Sato, M. & Kidera, A. Protein structural change upon ligand binding: Linear response theory. *Phys. Rev. Lett.* **94**, 078102 (2005).
