# Supplementary material for: Neural relational inference to learn long-range allosteric interactions in proteins from molecular dynamics simulations
Source: Nat Commun. 2022 Mar 29;13:1661. doi: 10.1038/s41467-022-29331-3 (PMC8964751; doi:10.1038/s41467-022-29331-3)
Supplement: Supplementary file 3 — Description of Additional Supplementary Files [file 41467_2022_29331_MOESM3_ESM.pdf]

Description of Additional Supplementary Files

File Name: Supplementary Movie 1
Description: Comparison between the actual simulation trajectories and the reconstructions for apo Pin1, FFpSPR-bound Pin1, and FFpSPR-bound Pin1 (I28A) (PDB ID: 3TDB).

File Name: Supplementary Movie 2
Description: Comparison between the actual simulation trajectories and the reconstructions for FFpSPR-bound Pin1 and pCdc25Cbound Pin1 (PDB ID: 1NMV).

File Name: Supplementary Movie 3
Description: Comparison between the actual simulation trajectories and the reconstructions for WT and G93A of SOD1.

File Name: Supplementary Movie 4
Description: Comparison between the actual simulation trajectories for WT, A52V, S218Sp/S222Sp, and E203K of MEK1.
