# Supplementary material for: Neural relational inference to learn long-range allosteric interactions in proteins from molecular dynamics simulations
Source: Nat Commun. 2022 Mar 29;13:1661. doi: 10.1038/s41467-022-29331-3 (PMC8964751; doi:10.1038/s41467-022-29331-3)
Supplement: Supplementary file 5 — Reporting Summary [file 41467_2022_29331_MOESM5_ESM.pdf]

nature portfolio

Corresponding author(s): Weiwei Han & Dong Xu

Last updated by author(s): Mar 2, 2022

Reporting Summary

Nature Portfolio wishes to improve the reproducibility of the work that we publish. This form provides structure for consistency and transparency in reporting. For further information on Nature Portfolio policies, see our Editorial Policies and the Editorial Policy Checklist.

Statistics

For all statistical analyses, confirm that the following items are present in the figure legend, table legend, main text, or Methods section.

n/a	Confirmed	
☐	☒	The exact sample size (*n*) for each experimental group/condition, given as a discrete number and unit of measurement
☐	☒	A statement on whether measurements were taken from distinct samples or whether the same sample was measured repeatedly
☐	☒	The statistical test(s) used AND whether they are one- or two-sided<br>*Only common tests should be described solely by name; describe more complex techniques in the Methods section.*
☐	☒	A description of all covariates tested
☐	☒	A description of any assumptions or corrections, such as tests of normality and adjustment for multiple comparisons
☐	☒	A full description of the statistical parameters including central tendency (e.g. means) or other basic estimates (e.g. regression coefficient) AND variation (e.g. standard deviation) or associated estimates of uncertainty (e.g. confidence intervals)
☐	☒	For null hypothesis testing, the test statistic (e.g. *F*, *t*, *r*) with confidence intervals, effect sizes, degrees of freedom and *P* value noted<br>*Give P values as exact values whenever suitable.*
☒	☐	For Bayesian analysis, information on the choice of priors and Markov chain Monte Carlo settings
☒	☐	For hierarchical and complex designs, identification of the appropriate level for tests and full reporting of outcomes
☐	☒	Estimates of effect sizes (e.g. Cohen's *d*, Pearson's *r*), indicating how they were calculated

Our web collection on statistics for biologists contains articles on many of the points above.

Software and code

Policy information about availability of computer code

Data collection	Amber-18-cuda-8.0, GROMACS 5.1.4 package
Data analysis	NRI-MD is open source and publicly available at https://github.com/juexinwang/NRI-MD. Other Tools and packages used in this paper include: Python version 3.7.1, torch version 1.2, matplotlib version 3.1.1, seaborn version 0.9.0, numpy version 1.19.5, networkx version 2.3, argparse version 1.1, pandas version 0.25.1, R version 3.6.2, CNA version 2.0, VMD 1.9.3, AmberTools16, CPPTRAJ version 16.16, Cytoscape version 3.8.0, Autodock 4.2, and SWISS-MODEL server (https://swissmodel.expasy.org/).

For manuscripts utilizing custom algorithms or software that are central to the research but not yet described in published literature, software must be made available to editors and reviewers. We strongly encourage code deposition in a community repository (e.g. GitHub). See the Nature Portfolio guidelines for submitting code & software for further information.

Data

Policy information about availability of data

All manuscripts must include a data availability statement. This statement should provide the following information, where applicable:

- Accession codes, unique identifiers, or web links for publicly available datasets
- A description of any restrictions on data availability
- For clinical datasets or third party data, please ensure that the statement adheres to our policy

The MD simulations used in the current study are publicly available. Three case studies data sets have been deposited in the Zenodo database (https://doi.org/10.5281/zenodo.5941385). The initial structures of Pin1 system obtained from PDB 3TDB (http://doi.org/10.2210/pdb3TDB/pdb), 1PIN (http://doi.org/10.2210/pdb1PIN/pdb), and 1NMV (http://doi.org/10.2210/pdb1NMV/pdb). The initial structures of SOD1 system obtained from PDB 2C9V (http://doi.org/10.2210/pdb2C9V/pdb). The initial structures of MEK1 system obtained from PDB 3SLS (http://doi.org/10.2210/pdb3SLS/pdb). All the analysis data

supporting the finding of this study are provided in the Source data.

Field-specific reporting

Please select the one below that is the best fit for your research. If you are not sure, read the appropriate sections before making your selection.

- [x] Life sciences
- [ ] Behavioural & social sciences
- [ ] Ecological, evolutionary & environmental sciences

For a reference copy of the document with all sections, see nature.com/documents/nr-reporting-summary-flat.pdf

Life sciences study design

All studies must disclose on these points even when the disclosure is negative.

Sample size	We ran the MD simulations for three case studies. Specifically, For the closed Pin1 structures (Apo-Pin1, FFpSPR-Pin1, and FFpSPR-Pin1(I28A) obtained from PDB 3TDB), the first simulation was run 200 ns, and the last two simulations were run 500 ns. For the open Pin1 structures (FFpSPR-Pin1 and pCdc25C-Pin1 obtained from PDB 1NMV), all three simulations were run 500 ns. For the closed pCdc25C-Pin1 structure (obtained from PDB 1PIN), the simulation was run 500 ns. For the wild type (WT) and 23 Ala-mutants of unbound Pin1 (obtained from PDB 1PIN), all the simulations were run 500 ns. For the SOD1 system, the first simulation was run 300 ns, the last two simulations were run 500 ns. For the MEK1 system, the first simulation was run 200 ns, the last two simulations were run 1 us.
Data exclusions	No
Replication	The MD simulations for three case studies were performed three times. All attempts at replication were successful.
Randomization	No
Blinding	No

Reporting for specific materials, systems and methods

We require information from authors about some types of materials, experimental systems and methods used in many studies. Here, indicate whether each material, system or method listed is relevant to your study. If you are not sure if a list item applies to your research, read the appropriate section before selecting a response.

Materials & experimental systems

n/a	Involved in the study	
x		Antibodies
x		Eukaryotic cell lines
x		Palaeontology and archaeology
x		Animals and other organisms
x		Human research participants
x		Clinical data
x		Dual use research of concern

Methods

n/a	Involved in the study	
x		ChIP-seq
x		Flow cytometry
x		MRI-based neuroimaging
